# Ethnobotanical Survey of Natural Galactagogues Prescribed in Traditional Chinese Medicine Pharmacies in Taiwan

**DOI:** 10.3389/fphar.2020.625869

**Published:** 2021-02-12

**Authors:** Jung Chao, Chien-Yu Ko, Chin-Yu Lin, Maeda Tomoji, Chia-Hung Huang, Hung-Che Chiang, Jeng-Jer Yang, Shyh-Shyun Huang, Shan-Yu Su

**Affiliations:** ^1^ Department of Chinese Pharmaceutical Sciences and Chinese Medicine Resources, Chinese Medicine Research Center, China Medical University, Taichung, Taiwan; ^2^ School of Pharmacy, China Medical University, Taichung, Taiwan; ^3^ Institute of New Drug Development, China Medical University, Taichung, Taiwan; ^4^ Tsuzuki Institute for Traditional Medicine, China Medical University, Taichung, Taiwan; ^5^ Department of Pharmaceutical Sciences, Nihon Pharmaceutical University, Saitama, Japan; ^6^ Department of Pharmacy, Kinmen Hospital, Kinmen, Taiwan; ^7^ College of Medicine, China Medical University, Taichung, Taiwan; ^8^ Department of Pharmacy, Chia Nan University of Pharmacy and Science, Tainan, Taiwan; ^9^ Department of Food Nutrition and Health Biotechnology, Asia University, Taichung, Taiwan; ^10^ Department of Chinese Medicine, China Medical University Hospital, Taichung, Taiwan; ^11^ School of Post-Baccalaureate Chinese Medicine, College of Chinese Medicine, China Medical University, Taichung, Taiwan

**Keywords:** breastfeeding, ethnobotanical, galactagogues, Taiwan, traditional Chinese medicine pharmacy

## Abstract

Natural medicinal materials have been used to promote breast milk secretion. Here, we investigated the natural medicinal materials prescribed in traditional Chinese medicine (TCM) pharmacies across Taiwan to induce lactation. We collected medicinal materials from 87 TCM pharmacies, identified them in the prescriptions, and analyzed their drug contents. We examined their botanical origins, biological classifications, traditional usage, and modern pharmacological properties. We used the TCM Inheritance Support System to identify core medicinal materials in galactogenous prescriptions. We collected 81 medicinal materials from 90 galactogenous prescriptions. *Leguminosae* accounted for 12%, whereas *Apiaceae* accounted for 7% of all materials examined. The primary medicinal plant parts used were roots and seeds. Nineteen frequently used medicinal materials had a relative frequency of citation of greater than or equal to 0.2. According to their efficacy, 58% were warm, 54% were sweet, and 63% were tonifying; 74% of the frequently used medicinal materials have been showed efficacy against breast cancer. The primary core medicinal material was *Angelica sinensis* (Oliv.) Diels, whereas the secondary core medicinal materials were *Tetrapanax papyrifer* (Hook.) K. Koch and *Hedysarum polybotrys* Hand.-Mazz. Most galactogenous prescriptions consisted of multiple materials from *Leguminosae* and *Apiaceae*. The mechanisms underlying galactogenous efficacy warrant further investigations.

## Introduction

Breast milk is rich in proteins, lipids, carbohydrates, vitamins, and minerals, making it the optimum nutrient source for infant growth and development (Suzuki et al., 1972). Breastfeeding prevents the death of approximately 823,000 children aged less than 5 years annually. Moreover, the incidence of breast and ovarian cancers in women who breastfed their children is 7% and 35% lower than those who never breastfed, respectively (Victora et al., 2016). Therefore, the World Health Organization (WHO) and the American Academy of Pediatrics recommend that infants under 6 months of age should be exclusively breastfed (Eidelman, 2012; World Health Organization, 2017). However, the subsequent investigations have revealed that only 40% of all infants worldwide are exclusively breastfed (World Health Organization, 2020). Hence, the WHO set a target to increase the exclusive breastfeeding rate within the first 6 months by more than 50% by 2025 (World Health Organization, 2013).

Insufficient post-delivery milk secretion is due to the fact that many women cannot exclusively breastfeed their newborns. A US survey showed that 76% of all mothers do not produce sufficient breast milk to meet the nutritional requirements of their babies (Bazzano et al., 2017). Galactagogues are used to increase breast milk secretion in mothers who intend to breastfeed their newborns exclusively. The two primary categories of galactagogues are pharmaceutical agents and herbs. In Western medicine, galactogenous pharmaceutical agents with a high efficacy, such as metoclopramide, domperidone, and chlorpromazine, are widely used as galactagogues (Foong et al., 2020). Furthermore, herbs have been used to promote lactation in various parts of the world (Mortel and Mehta, 2013; Özalkaya et al., 2018). These medicinal materials significantly vary among regions, customs, and religious traditions. Medicinal plant materials that are most frequently used as natural galactagogues include *Trigonella foenum-graecum* L. (fenugreek) and *Foeniculum vulgare* Mill. (fennel), which are used in the United States, Australia, and China to promote milk production (Supplementary Table S1).

The “doing-the-month (one-month puerperal care)” custom is practiced in several countries that have a Chinese population (Liu Y. Q. et al., 2015), including Taiwan. In this custom, medicinal materials are routinely used to enhance physical recovery and increase milk secretion in puerperal mothers (Chen and Wang, 2000; Chuang et al., 2009; Tsai and Wang, 2019). This custom may account for the higher proportion of exclusively breastfed infants (46.3%) than the average for other parts of the world (Ministry of Health and Welfare, 2020). Moreover, the proportion of Taiwanese women with self-perceived milk insufficiency (nearly 50%) is lower than that reported for mothers in other parts of the world (Su, 2012). According to a previous survey, approximately 80% of all Chinese herbal medicines used by Taiwanese women during their puerperal period were purchased from traditional Chinese medicine (TCM) pharmacies (Ho et al., 2011). TCM pharmacies provide TCM formulae in various dosage forms including pills, powders, paste, pellets, and decoction pieces, and preserve the original forms of a TCM (Pharmaceutical Affairs Act, 2018). Most TCMs consumed by Taiwanese women following delivery are in the form of decoction pieces (Ho et al., 2011). Hence, in the present study, we selected TCM pharmacies as the primary investigation sites to clarify the current use of Chinese herbal galactogenous prescriptions by Taiwanese women.

In Taiwan, although “galactogenous prescriptions” may be procured from most TCM pharmacies, the prescriptions differ among TCM pharmacies in medicinal materials. To date, no study has investigated the types and combinations of medicinal materials in galactogenous prescriptions. Thus, the aims of this study were as follows: to 1) explore the compositions of galactogenous prescriptions sold in TCM pharmacies, 2) systematically analyze them, 3) identify their core components, and 4) elucidate the principles and preparation methods of TCM that are used to promote lactation in post-delivery Taiwanese women.

## Materials and Methods

### Ethical Review

This research was conducted from July 2019 to May 2020 and was reviewed and approved by the China Medical University & Hospital Research Ethics Center (No. CRREC-108-026) (Supplementary Figure S1).

### Research Process

**Figure 1:**
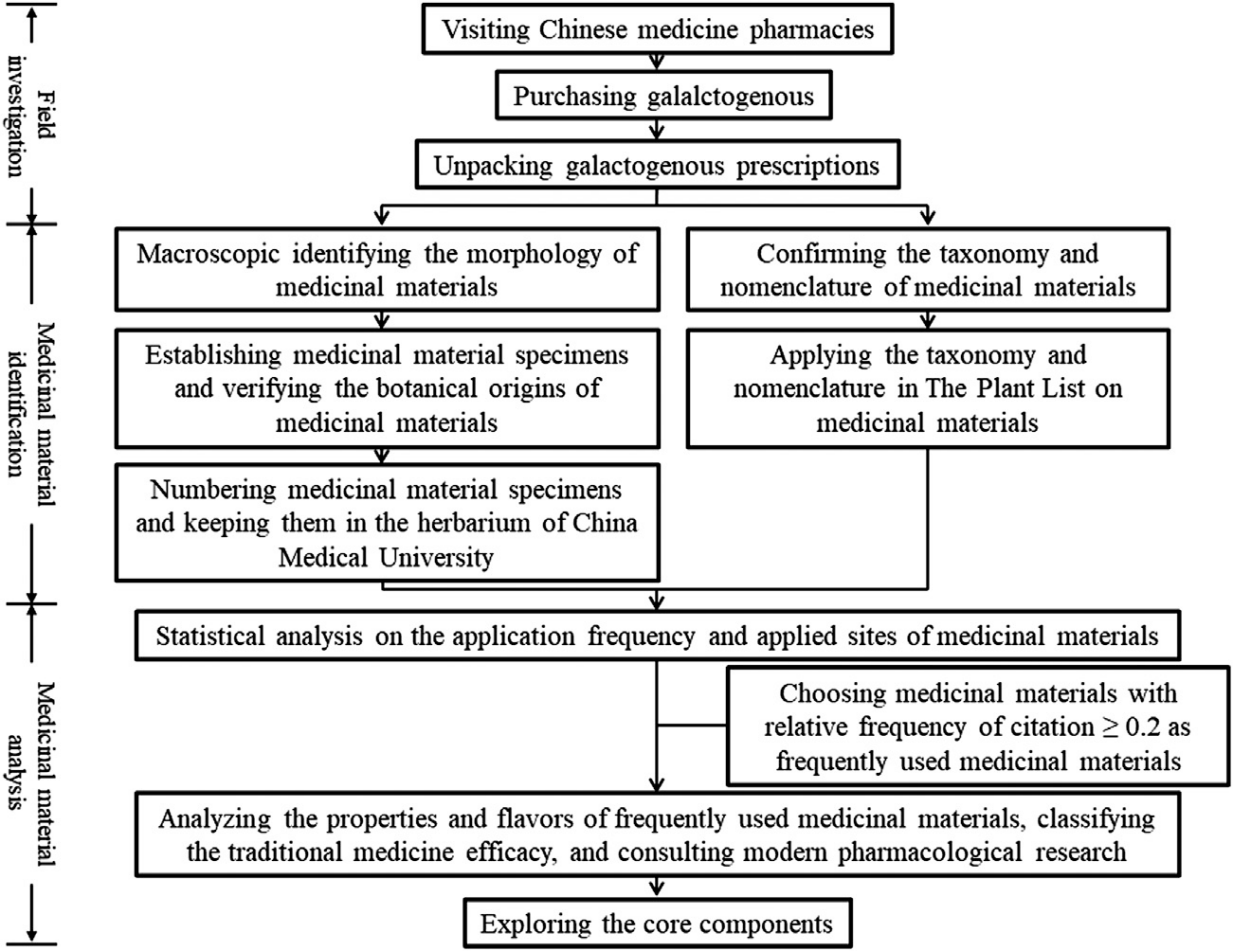
Research flowchart.

The research methods are summarized in the research flow chart (Figure 1). This study involved field investigation, medicinal material identification, and medicinal material analysis.

#### Field Investigation

**Figure 2:**
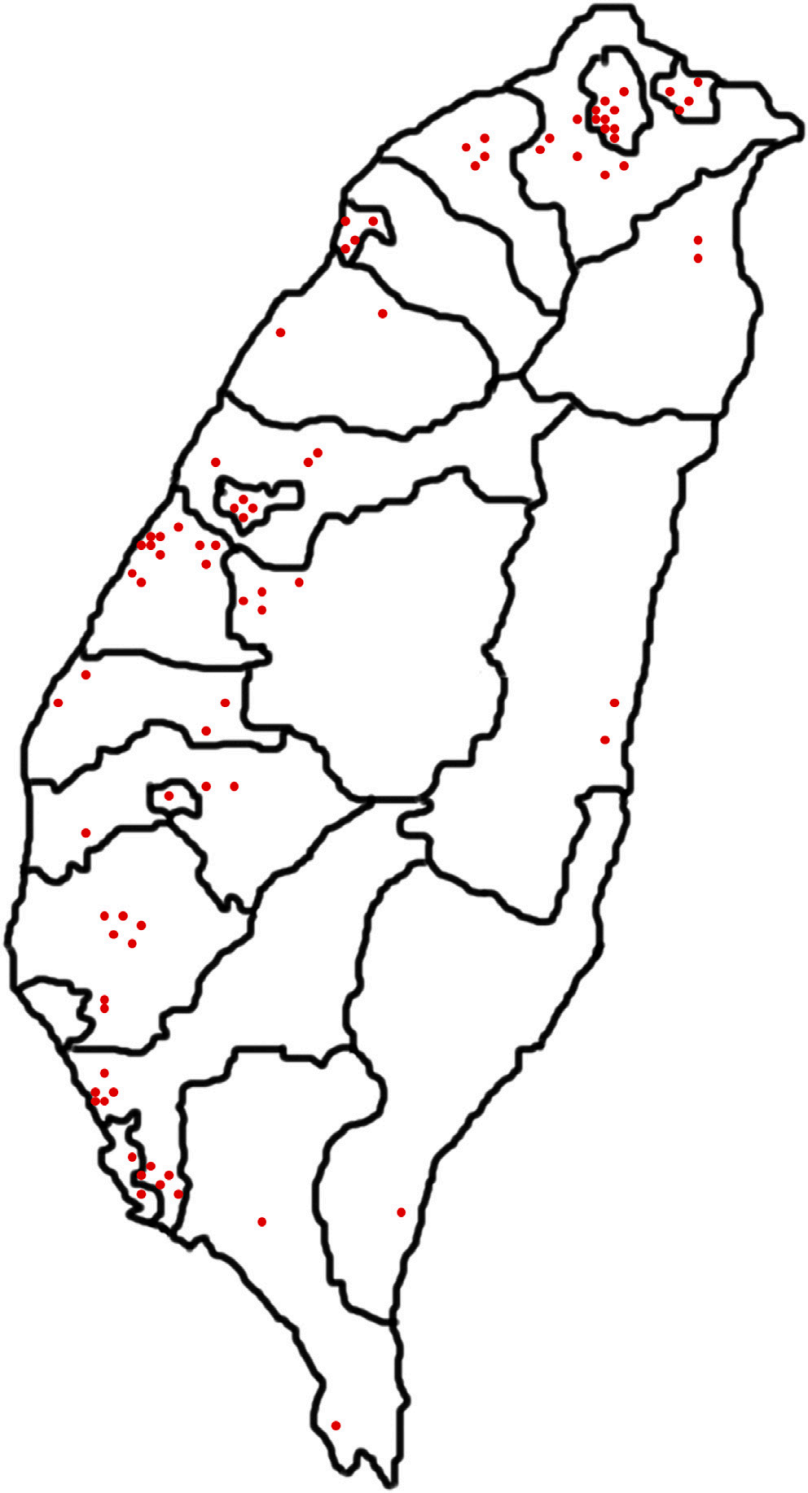
Geographical distribution of stores in Taiwan where galactogenous prescriptions were purchased.

Taiwan is an island in East Asia located at 21°45′–25°56′N and 119°18′–124°34′E, covering an area of 35,886.8623 km^2^. The Tropic of *Cancer* passes through it, and its climate is Humid Subtropical according to the Köppen Classification. This study lasted 12 months, from May 2019 to April 2020. Eighty-seven TCM pharmacies providing galactogenous prescriptions were visited (Figure 2). The relative numbers of pharmacies visited were proportional to the population ratio of each city and county. The outlets were located via online searches and various organizations associated with medicinal plants. Ninety galactogenous prescriptions were obtained. The TCM pharmacies investigated were distributed across northern, central, southern, and eastern Taiwan. As the Taiwanese population density is uneven, comparatively more samples were collected in the western part of the island. Each area where a TCM pharmacy was located had its own characteristic demographics, planting patterns, Chinese herbal medicine distribution, economic development level, and geography (Supplementary Table S2).

#### Analysis of Medicinal Materials

All investigated medicinal materials were analyzed in terms of taxonomy, relative frequency of citation (RFC), inclusion status in each pharmacopoeia, modern pharmacological research related to application in women, and efficacy in traditional medicine.

Taxonomy comprised the scientific, kingdom, and family names and utilized parts. The information was derived from The Plant List (The Royal Botanic Gardens, 2013). Medicinal materials with an RFC of greater than or equal to 0.2 were defined as frequently used medicinal materials. RFC was calculated as follows (Ahmad et al., 2017):

For the inclusion status of the medicinal materials in the pharmacopoeia, the Third Edition of the Taiwan Herbal Pharmacopeia (Chen, 2018), the Pharmacopoeia of the People’s Republic of China (Chinese Pharmacopoeia Commission, 2020), and the Chinese Materia Medica (State Administration of TCM, 1999) were verified. Modern pharmacological studies related to women were searched and identified using PubMed by entering the scientific names of the medicinal materials as keywords and setting the sex as female and the inquiry period as 1992–2020. The traditional efficacy, property, and flavor of the medicinal materials were cited according to the records in the Taiwan Herbal Pharmacopeia, the Pharmacopoeia of the People’s Republic of China, and the Chinese Materia Medica.

The TCM Inheritance Support System (TCMISS) v. 2.5 conducted the network, composition, and correlation analyses. This system was designed to mine core Chinese material medica (CCMM) and visualize correlations based on nodes and links. The same materials may have different names; hence, the nomenclature was standardized for data input (Wu et al., 2020). With respect to composition setting for the network analysis, the frequency of occurrence of a medicinal material increased with a decrease in distance from the center of the network diagram. Thus, medicinal materials nearest to the center served as references to determine the core components of galactogenous prescriptions. When two medicinal materials co-occurred more than 41 times in the composition setting for the network analysis, they were considered as a high-frequency drug pair. Application frequency and confidence score were set, and the former was calculated as follows:

In the correlation analysis, when two different medicinal materials co-appeared more than 18 times, they were considered to be correlated and were connected by a line in the network diagram. The correlation analysis diagram was associated with the confidence level, which indicates the probability that a medicinal material co-occurs with another one. When the TCMISS was used to analyze the correlation among the medicinal materials used, the confidence was set to unity; in this way, medicinal materials that co-occurred with others were identified (Tang et al., 2019; Wu et al., 2019).

## Results

### Types and Taxonomic Characteristics of Galactogenous Prescriptions

**Figure 3:**
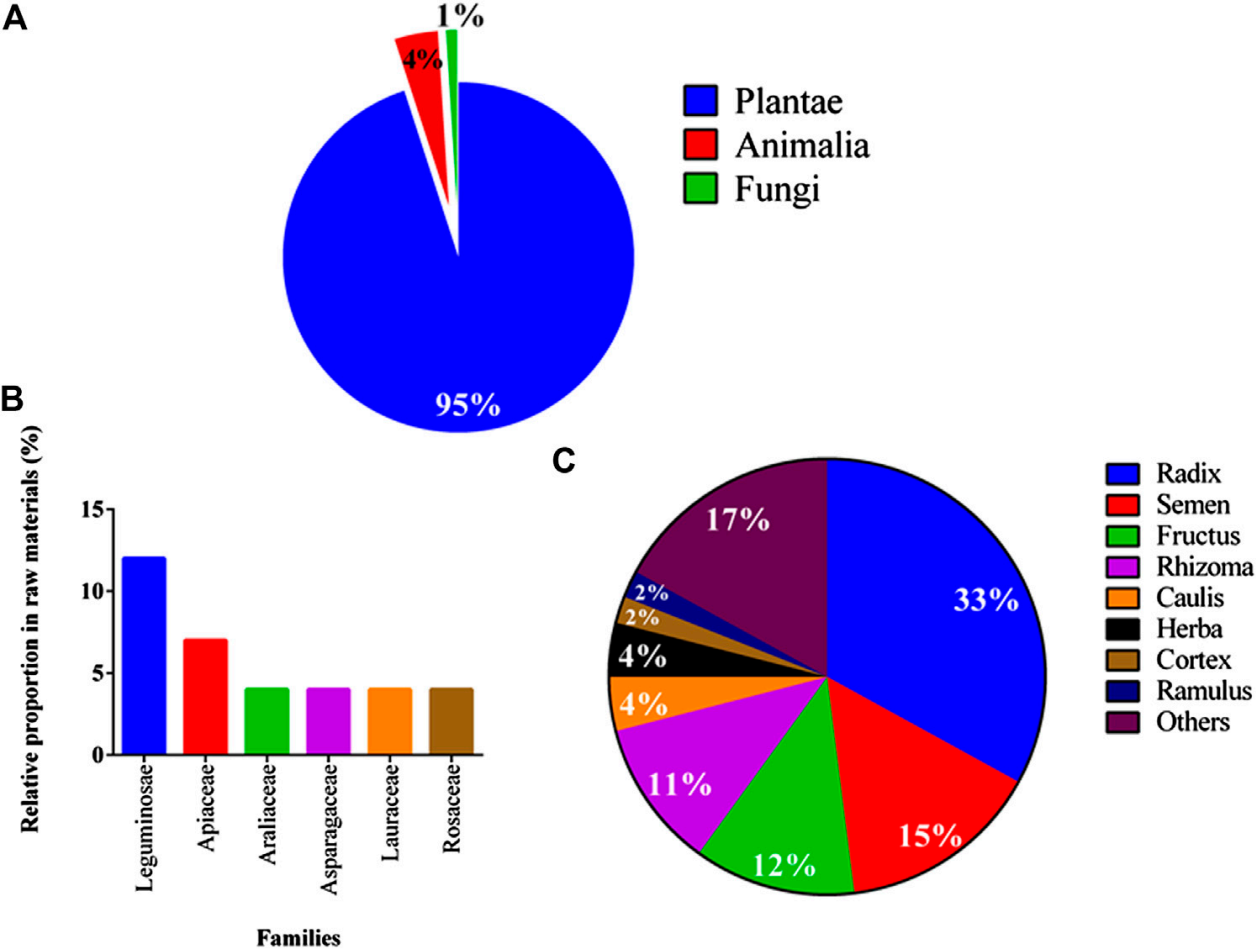
Taxonomy of 81 medicinal materials in 90 galactogenous prescriptions. **(A)** Kingdoms, **(B)** families, and **(C)** utilized parts. Parts collectively used less than 1% are summarized as “others.”

Eighty-seven TCM pharmacies were visited in various cities and counties in Taiwan and 90 galactogenous prescriptions were purchased (Supplementary Figure S2); 81 medicinal materials were identified (Supplementary Table S3). Seventy-eight medicinal materials were plant based (95%), two were animal derived (4%), and one was a fungus (1%). *Angelica sinensis* (Oliv.) Diels was the most frequently used medicinal material (93%), followed by *Tetrapanax papyrifer* (Hook.) K. Koch (86%). *Leguminosae* members (12%) were the most frequently used, followed by *Apiaceae* members (7%). The roots (radix) were the most frequently utilized plant parts (33%), followed by the seeds (15%; Figure 3).

### Traditional Efficacy and Modern Pharmacological Analyses of Medicinal Materials Frequently Used in Galactogenous Prescriptions

**Figure 4:**
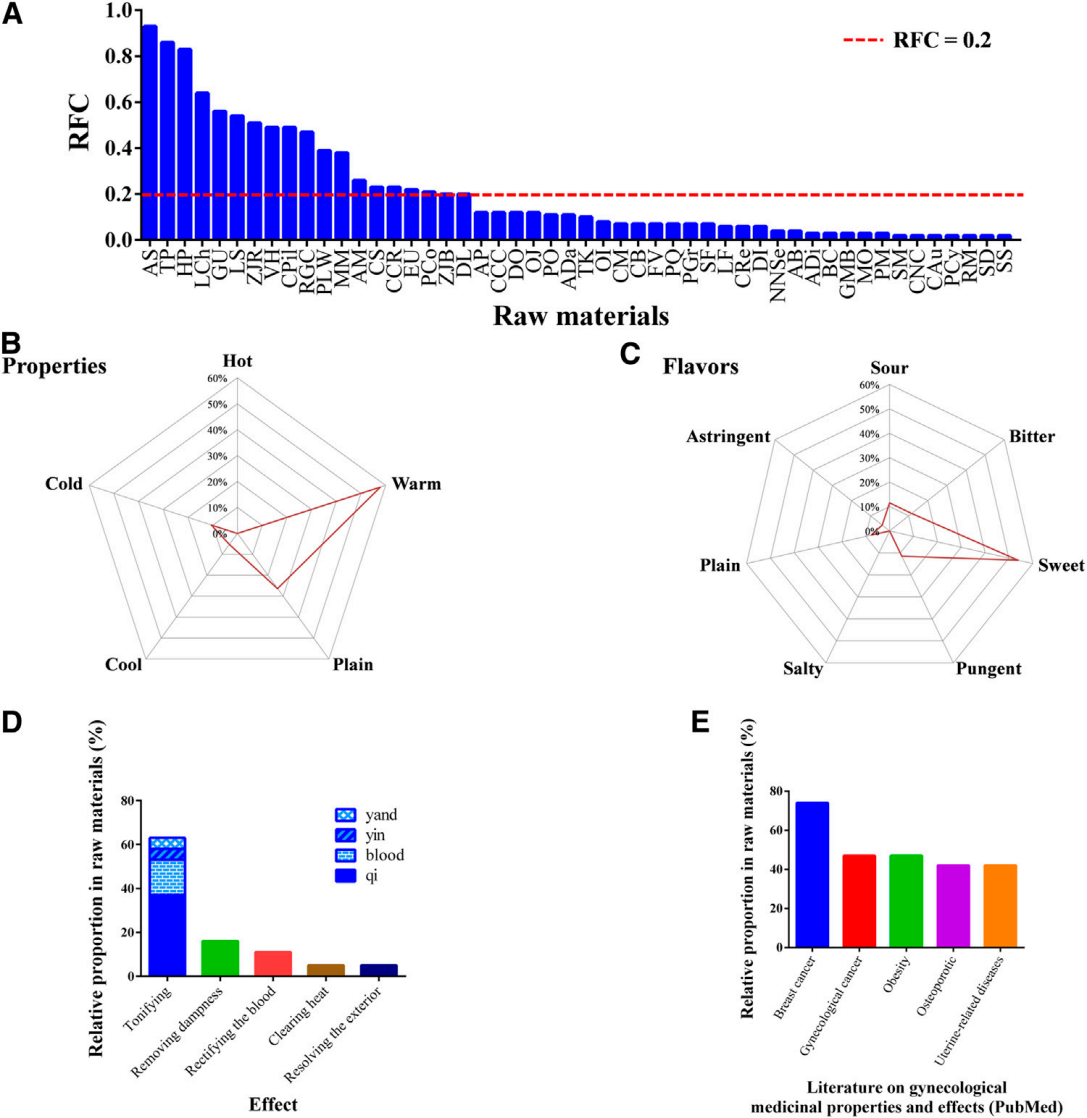
Characteristics of 19 medicinal materials with an RFC of greater than or equal to 0.2 in galactogenous prescriptions. **(A)** Selection of frequently used medicinal materials. **(B)** Radar chart of properties. **(C)** Radar chart of flavors. **(D)** Histogram of traditional efficacy classifications. **(E)** Modern pharmacological research related to women. RFC, relative frequency of citation; AB, *Achyranthes bidentata* Blume; ADa, *Angelica dahurica* (Hoffm.) Benth. and Hook. f. ex Franch. and Sav.; ADi, *Araiostegia divaricata* (Blume) M. Kato; AM, *Atractylodes macrocephala* Koidz.; AP, *Astragalus propinquus* Schischkin; AS, *Angelica sinensis* (Oliv.) Diels; BC, *Bupleurum chinense* DC.; CAu, *Cuscuta australis* R. Br.; CB, *Cibotium barometz* (L.) J. Sm.; CCC, *Cinnamomum cassia* (L.) J. Presl (cortex); CCR, *Cinnamomum cassia* (L.) J. Presl (ramulus); CM, *Clematis montana* Buch.-Ham. ex DC.; CNC, *Cervus nippon* Temminck (cornupantotrichum); CPil: *Codonopsis pilosula* (Franch.) Nannf.; CRe: *Citrus reticulata* Blanco; CS, *Chaenomeles speciosa* (Sweet) Nakai; DI, *Dipsacus inermis* Wall.; DL, *Dimocarpus longan* Lour.; DO, *Dioscorea oppositifolia* L.; EU: *Eucommia ulmoides* Oliv.; FV, *Foeniculum vulgare* Mill.; GMB, *Glycine* max (L.) Merr. (black); GU, *Glycyrrhiza uralensis* Fisch.; HP, *Hedysarum polybotrys* Hand.-Mazz.; LCh, *Lycium chinense* Mill.; LF, *Liquidambar formosana* Hance; LS, *Ligusticum striatum* DC.; MM, *Melastoma malabathricum* L.; MO, *Morinda officinalis* F.C.How; NNSe, *Nelumbo nucifera* Gaertn. (Semen); OI, *Oroxylum indicum* (L.) Kurz; OJ, *Ophiopogon japonicus* (Thunb.) Ker Gawl.; PCo, *Poria cocos* (Schwein.) F.A. Wolf; PCy, *Polygonatum cyrtonema* Hua; PGr, *Platycodon grandiflorus* (Jacq.) A. DC.; PLW, *Paeonia lactiflora* Pall. (white); PM, *Pueraria montana* var. lobata (Willd.) Sanjappa & Pradeep; PO, *Polygonatum odoratum* (Mill.) Druce; PQ, *Panaxquin quefolius* L.; RGC, *Rehmannia glutinosa* (Gaertn.) DC. (cooked); RM, *Reynoutria multiflora* (Thunb.) Moldenke; SF, *Strobilanthes forrestii* Diels; SM, *Salvia miltiorrhiza* Bunge; TK, *Trichosanthes kirilowii* Maxim.; TP, *Tetrapanax papyrifer* (Hook.) K. Koch; VH, *Vaccaria hispanica* (Mill.) Rauschert; ZJB, *Ziziphus jujuba* Mill. (black); ZJR, *Ziziphus jujuba* Mill. (red).

The RFC of 0.2 was set as the cutoff for frequently used medicinal materials, and accordingly, 19 medicinal materialswere identified among the 81 medicinal materials (Table 1). To understand the efficacy of medicinal materials in traditional medicine and the modern pharmacology of Chinese herbal medicine in promoting lactation, we analyzed the property, flavor, efficacy, and modern pharmacology research of these frequently used medicinal materials in women (Figure 4A).

**TABLE 1:** Medicinal properties of materials frequently used in galactogenous prescriptions (RFC ≥ 0.2) and modern pharmacological research on their applications for women.

No	Scientific name/local name	Family	Part used	RFC^a^	Flavor and property	Traditional usage	Literature on gynecological medicinal properties and effects (PubMed)
1	*Angelica sinensis* (oliv.) diels/Tang kuei	*Apiaceae*	Radix	0.93	Sweet and pungent; warm	Enriching blood and promoting blood circulation, regulating and alleviating menstruation pain, lubricating intestines, and relieving constipation	1. Anemia Chang et al. (2016), Chen et al. (2018), Li et al. (2012), Liu J. et al. (2019), and Zhang W.L. et al. (2012) 2. Blood stasis syndrome Jin et al. (2017), and Yuan et al. (2019) 3. Breast cancer He et al. (1986), Rock and DeMichele (2003), Zhou et al. (2015), Lin et al. (2017), Ma et al. (2017), Qi et al. (2017), and Su et al. (2018) 4. Female reproductive problems Du et al. (2014), Hook (2014), Gong et al. (2016) 5. Female sexual dysfunction Mazaro-Costa et al. (2010) 6. Gynecological cancer^b^ Cao et al. (2010), and Lang et al. (2018) 7. Hair loss Kim et al. (2014) 8. Mastitis Wang et al. (2012), and Mullen et al. (2014) 9. Obesity Zhong et al. (2017) 10. Osteoporotic Rock and DeMichele (2003), Xie et al. (2012), Lim and Kim (2014), and Li et al. (2016) 11. Puerperal metritis Huang et al. (2018)
2	*Tetrapanax papyrifer* (Hook.) K. Koch/T’ung ts’ao	*Araliaceae*	Medulla	0.86	Sweet and plain; cold	Clearing heat, promoting urination, dredging *qi*, and promoting lactation	None
3	*Hedysarum polybotrys* Hand.-Mazz./Hung ch’I	*Leguminosae*	Radix	0.83	Sweet; warm	Tonifying *qi,* lifting yang, consolidating exterior, reducing sweat, promoting urination, alleviating edema, regenerating body fluids, nourishing blood, activating stagnation, alleviating arthralgia, eliminating toxins, expelling pus, healing sores, and promoting granulation	None
4	*Lycium chinense* Mill./Kou ch’I	*Solanaceae*	Fructus	0.64	Sweet; plain	Nourishing liver and kidneys, enriching essence, and improving eyesight	1. Breast cancer Li et al. (2009), Zhang et al. (2011), Wawruszak et al. (2016), Georgiev et al. (2019) 2. Endometrial damage Lee et al. (2016), Shan et al. (2017) 3. Gynecological cancer Zhang et al. (2011) 4. Obesity Amagase and Nance (2011), de Souza Zanchet et al. (2017), Kim et al. (2017a) 5. Osteoporotic Yin et al. (2004), Kim et al. (2017b) 6. Ovarian injury Wei et al. (2011), Yang D.M. et al. (2017) 7. Polycysticovarian syndrome Jang et al. (2014) 8. Premature ovarian failure Chao et al. (2003)
5	*Glycyrrhiza uralensis* Fisch./Kan ts’ao	*Leguminosae*	Radix	0.56	Sweet; plain	Invigorating spleen, enriching *qi*; clearing heat; removing toxicity; resolving phlegm; relieving cough, spasm, and pain; and coordinating mechanisms of several medicinal materials simultaneously	1. Breast cancer Hu et al. (2009), Seon et al. (2012), Park et al. (2016), Huang et al. (2019) 2. Female reproductive problems Hajirahimkhan et al. (2013), Jia et al. (2013), Arentz et al. (2014), Hajirahimkhan et al. (2015) 3. Gynecological cancer Liu et al. (2017) 4. Obesity Lee H. E. et al. (2018) 5. Polycystic ovarian syndrome Arentz et al. (2014) 6. Puerperal metritis Huang et al. (2018) 7. Uterine contraction Yang L. et al. (2017)
6	*Ligusticum striatum* DC/Ch’uan ch’iung	*Apiaceae*	Rhizoma	0.54	Pungent; warm	Activating blood and *qi* circulation, expelling wind, and relieving pain	1. Anemia Li et al. (2012) 2. Puerperal metritis Huang et al. (2018)
7	*Ziziphus jujuba* Mill./Hung tsao	*Rhamnaceae*	Fructus	0.51	Sweet; warm	Strengthening middle warmer and enriching *qi*, nourishing blood, and calming nerves	1. Breast cancer Plastina et al. (2012) 2. Gynecological cancer Tahergorabi et al. (2015) 3. Hair loss Yoon et al. (2010) 4. Obesity Tahergorabi et al. (2015), Kawabata et al. (2017)
8	*Vaccaria hispanica* (Mill.) Rauschert/Wang pu liu hsing)	*Caryophyllaceae*	Semen	0.49	Bitter; plain	Activating blood circulation, unblocking menstrual flow, promoting lactation, reducing swelling, promoting urination, and treating stranguria	1. Breast cancer Shoemaker et al. (2005) 2. Milk synthesis Yu et al. (2020) 3. Osteoporotic Shih et al. (2009)
9	*Codonopsis pilosula* (Franch.) Nannf./Tang san	*Campanulaceae*	Radix	0.49	Sweet; plain	Invigorating spleen, ameliorating lungs, nourishing blood, and regenerating body fluids	1. Breast cancer Wang et al. (2014), Fu et al. (2016)
10	*Rehmannia glutinosa* (Gaertn.) DC./Shu ti huang	*Plantaginaceae*	Radix	0.47	Sweet; warm	Enriching blood, nourishing *yin*, enriching essence, and replenishing marrow	1. Anemia Liang et al. (2004) 2. Blood stasis syndrome Kubo et al. (1994) 3. Endometrial abnormality Lee et al. (2016) 4. Breast cancer Li et al. (2014), Liu C. et al. (2015) 5. Hair loss Lee C.Y. et al. (2018) 6. Obesity Han et al. (2015) 7. Osteoporotic Gong et al. (2019), Lai et al. (2015), Lim and Kim (2013), Liu C. et al. (2019), Oh et al. (2003), Ok et al. (2015), Yin et al. (2004) 8. Ovarian failure Chao et al. (2003), Wei et al. (2014) 9. Polycystic ovarian syndrome Liang et al. (2008), Jang et al. (2014)
11	*Paeonia lactiflora* Pall./Pai shao	*Paeoniaceae*	Radix	0.39	Bitter and sour; cold	Nourishing blood, regulating menstruation, making *yin* astringent, reducing sweat, softening liver, relieving pain, and suppressing hyperactive liver *yang*	1. Anemia Lee et al. (2014) 2. Blood stasis syndrome Sun et al. (2016), Cheng et al. (2018) 3. Breast cancer Liu Y.T. et al. (2019) 4. Female reproductive problems Arentz et al. (2014), Moini Jazani et al. (2018) 5. Menopausal hot flushes Li et al. (2019) 6. Osteoporotic Tsai et al. (2008) 7. Polycystic ovarian syndrome Arentz et al. (2014), Arentz et al. (2017) 8. Uterine myomas Sakamoto et al. (1992)
12	*Melastoma malabathricum* L./Yeh mu tan	*Melastomataceae*	Caulis & radix	0.38	Sour and astringent; cool	Removing retained food, promoting urination and blood circulation, stopping bleeding, clearing heat, and removing toxicity	1. Breast cancer Hamid et al. (2018)
13	*Atractylodes macrocephala* Koidz./Pai chu	*Compositae*	Rhizoma	0.26	Bitter and sweet; warm	Invigorating spleen, enriching *qi*, eliminating dampness, promoting urination, reducing sweat, and preventing miscarriage	1. Breast cancer Wang et al. (2014), Fu et al. (2016) 2. Gynecological cancer Long et al. (2017) 3. Obesity Song et al. (2018), Zhu et al. (2018) 4. Uterine contraction Zhang et al. (2000)
14	*Chaenomeles speciose* (sweet) Nakai/Mu kua	*Rosaceae*	Fructus	0.23	Sour; warm	Relaxing tendons, activating collaterals, harmonizing stomach, and eliminating dampness	None
15	*Cinnamomum cassia* (L.) J. Presl/Kuei chih	*Lauraceae*	Ramulus	0.23	Pungent and sweet; warm	Inducing perspiration, dispelling pathogenic factors from muscles, warming and dredging meridians, supporting yang, transforming into *qi*, suppressing upward surge of *qi*, and descending *qi*	1. Breast cancer Rad et al. (2015), Yu et al. (2019) 2. Gynecological cancer Koppikar et al. (2010) 3. Infertility Iwaoka et al. (2010) 4. Obesity Zhang et al. (2019) 5. Osteoporotic Huh et al. (2015) 6. Polycystic ovarian syndrome Arentz et al. (2014) 7. Uterine contraction Sun et al. (2016), Sun et al. (2017) 8. Uterine myomas Sakamoto et al. (1992)
16	*Eucommia ulmoides* Oliv./Tu chung	*Eucommiaceae*	Cortex	0.22	Sweet; warm	Tonifying liver and kidneys, strengthening bones and tendons, and preventing miscarriage	1. Obesity Zhang W. et al. (2012) 2. Osteoporotic Yin et al. (2004), Zhang et al. (2009), Zhang et al. (2014), Zhang W. et al. (2012) 3. Uterine contraction Ho et al. (2011)
17	*Poria cocos* (Schwein.) F.A. Wolf/Fu ling	*Polyporaceae*	Sclerotia	0.21	Sweet and plain; plain	Promoting urination, eliminating dampness, invigorating spleen, and calming heart	1. Anemia Shen et al. (2005) 2. Breast cancer Zhang et al. (2006), Ling et al. (2011) 3. Gynecological cancer Tao et al. (2016) 4. Osteoporotic Xia et al. (2014) 5. Polycystic ovarian syndrome Jang et al. (2014) 6. Uterine contraction Sun et al. (2016) 7. Uterine dysfunction Lee et al. (2016) 8. Uterinemyomas Sakamoto et al. (1992)
18	*Ziziphus jujuba* Mill./Hei tsao	*Rhamnaceae*	Fructus	0.20	Sweet; warm	Tonifying spleen and stomach, enriching *qi* and blood, calming heart and nerves, regulating *ying* and *wei*, and harmonizing medicinal properties of various ingredients simultaneously	1. Breast cancer Plastina et al. (2012) 2. Gynecological cancer Tahergorabi et al. (2015) 3. Hair loss Yoon et al. (2010) 4. Obesity Tahergorabi et al. (2015), Kawabata et al. (2017)
19	*Dimocarpus longan* Lour./Kuei yüan	*Sapindaceae*	Arillus	0.20	Sweet; warm	Tonifying heart and spleen, nourishing blood, and calming nerves	1. Breast cancer Khan et al. (2018) 2. Gynecological cancer Li et al. (2018)

^a^ RFC, relative frequency of citation.

^b^ Gynecological cancer includes cervical, ovarian, and uterine cancers.

The medicinal materials frequently used in galactogenous prescriptions are warm (58%) and plain (26%) in terms of property (Figure 4B). Regarding flavor, most of the medicinal materials used were sweet (54%) (Figure 4C). With respect to traditional medicine efficacy, most of these medicinal materials were tonics (63%) (Figure 4D). With respect to modern pharmacological effects related to women, the related studies have most frequently investigated anticancer efficacy. Fourteen medicinal materials (74%) among those with an RFC greater than or equal to 0.2 have been reported to be effective against breast cancer, whereas nine (47%) were effective against gynecological (cervical, ovarian, and uterine) cancers (Figure 4E).

### Analysis of High-Frequency Drug Pairs and Core Medicinal Materials

A TCMISS analysis disclosed 18 high-frequency drug pairs (Supplementary Table S4) and 2 medicinal materials that co-occurred more than 41 times including *A. sinensis*, *T. papyrifer*, *Hedysarum polybotrys* Hand.-Mazz., *Lycium chinense* Mill., *Glycyrrhiza uralensis* Fisch., *Ligusticum striatum* DC., *Ziziphus jujuba* Mill., *Vaccaria hispanica* (Mill.) Rauschert, *Codonopsis pilosula* (Franch.) Nannf., and *Rehmannia glutinosa* (Gaertn.) DC. The most frequently used drug pairs were *A. sinensis* plus *T. papyrifer* (frequency = 71) and *A. sinensis* plus *H. polybotrys* (frequency = 69).

**Figure 5:**
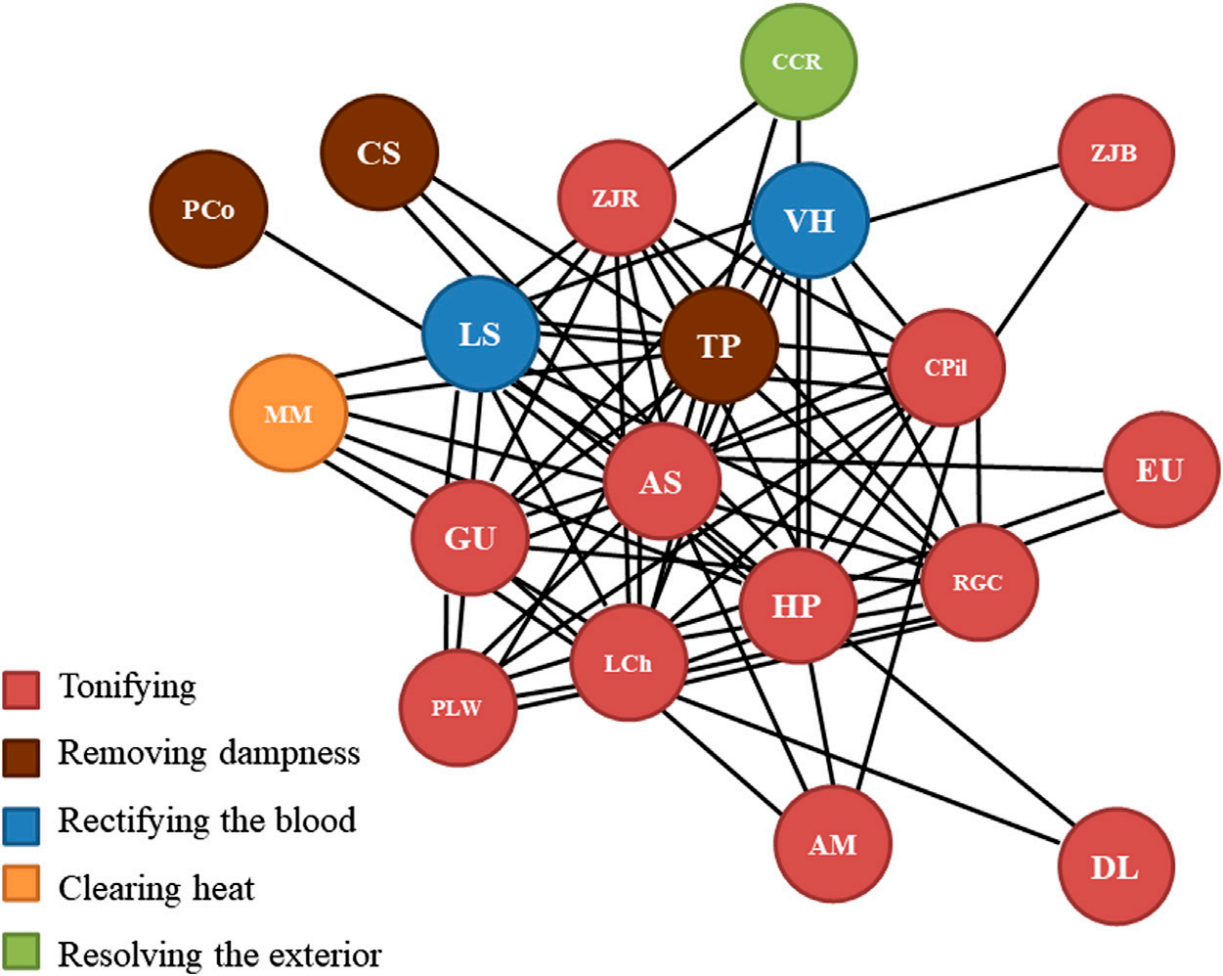
Network diagram representing the analysis of the core components in galactogenous prescriptions. AM, *Atractylodes macrocephala* Koidz.; AS, *Angelica sinensis* (Oliv.) Diels; CCR, *Cinnamomum cassia* (L.) J. Presl (ramulus); CPil, *Codonopsis pilosula* (Franch.) Nannf.; CS, *Chaenomeles speciosa* (Sweet) Nakai; DL, *Dimocarpus longan* Lour.; EU, *Eucommia ulmoides* Oliv.; GU, *Glycyrrhiza uralensis* Fisch.; HP, *Hedysarum polybotrys* Hand.-Mazz.; LCh, *Lycium chinense* Mill.; LS, *Ligusticum striatum* DC.; MM, *Melastoma malabathricum* L.; PCo, *Poria cocos* (Schwein.) F.A. Wolf; PLW, *Paeonia lactiflora* Pall. (white); RGC, *Rehmannia glutinosa* (Gaertn.) DC. (cooked); TP, *Tetrapanax papyrifer* (Hook.) K. Koch; VH, *Vaccaria hispanica* (Mill.) Rauschert; ZJB, *Ziziphus jujuba* Mill. (black); ZJR, *Ziziphus jujuba* Mill. (red).

A network analysis of the core components of the galactogenous prescriptions was conducted on medicinal materials with an RFC of greater than or equal to 0.2 (Figure 5). The top core medicinal materials were *A. sinensis*, followed by *T. papyrifer* and *H. polybotrys*. They were often co-prescribed with *L. chinense*, *G. uralensis*, *L. striatum*, *Z. jujuba*, *V. hispanica*, *C. pilosula*, *R. glutinosa*, *Paeonia lactiflora* Pall., and *Melastoma malabathricum* L. In certain prescriptions, *Atractylodes macrocephala* Koidz., *Chaenomeles speciosa* (Sweet) Nakai, *Cinnamomum cassia* (L.) J. Presl, *Eucommia ulmoides* Oliv., *Poria cocos* (Schwein.) F.A. Wolf, *Z. jujuba*, and *Dimocarpus longan* Lour. were added. These combinations may serve as a reference for a galactogenous prescription composition.

A causality analysis of the occurrence of various medicinal materials in the galactogenous prescriptions was conducted based on association rules (Supplementary Table S5). The confidence score was set to unity. *Angelica sinensis*, *T. papyrifer*, *H. polybotrys*, and *L. chinense* were often combined with *L. striatum*, *P. lactiflora*, *C. pilosula*, *C. cassia*, *V. hispanica*, *G. uralensis*, *Z. jujuba*, *A. macrocephala*, *E. ulmoides*, and *R. glutinosa*.

## Discussion

### Field Investigation Sites

In the present study, a field investigation was conducted to explore the galactogenous prescriptions sold in TCM pharmacies across Taiwan to reflect the views and behaviors of some individuals over a certain period. Field investigations are especially practical for sociological, geographical, and cultural studies (Hirsch and Stewart, 2005), and are used to examine medications administered for certain diseases in certain realms of ethnopharmacological research. Field investigations related to herbal medicines have been performed to explore the composition of herbal teas (Huang et al., 2020), herbal medicines used to treat malaria (Odoh et al., 2018), and regional herbal medicines prescribed to expel parasites (Bajin Ba Ndob et al., 2016).

The “TCM pharmacies” in Taiwan are important for preserving TCM culture. In early agricultural societies, Western medicine was underdeveloped and medical resources were inadequate; TCM pharmacies provided medical care. During the period when the Japanese occupied Taiwan (1895), purveyors of TCMs were called “TCM merchants” or “medicinal material merchants” (Chang, 1995), whereas today, they are generally called “TCM merchants” or “TCM pharmacists” (Legislative Yuan, 2000). Under the Japanese medical care administrative measures in Taiwan, “an attitude of abandoning traditional Chinese medical care but retaining traditional Chinese medications” was adopted and medicine merchants were not strictly regulated (Ministry of Science and Technology, 2017). Therefore, TCM pharmacies in Taiwanese society are continued as the “traditional Chinese pharmaceutical industry” and provide both medical care and health maintenance. The TCM pharmacies provide Chinese medicinal materials based on customer requirements. They also furnish traditional dosage forms including pills, powder, paste, and decoction pieces prepared according to the fixed formulae (Pharmaceutical Affairs Act, 2018). The medical insurance of the Taiwanese Government covers only extracted granules of Chinese medicinal medica. Consequently, many consumers and TCM practitioners are unfamiliar with Chinese medicinal materials. The TCM pharmacies visited in the present study focused primarily on decoction pieces. Thus, it was ascertained that the techniques used to prepare TCM decoction pieces have been preserved by the TCM pharmacies in Taiwan. The results of the current investigation reflect the current prescription status of Chinese herbal decoction pieces for promoting lactation in Taiwanese women.

### Types and Taxonomic Traits of Medicinal Materials in Galactogenous Prescriptions Sold in TCM Pharmacies Across Taiwan

Members of *Leguminosae* were the most frequently used medicinal materials in the galactogenous prescriptions across Taiwan, including *Z. jujuba* and *G. uralensis*, followed by *Apiaceae* members such as *A. sinensis* and *L. striatum*. Flavones are abundant in both *Leguminosae* and *Apiaceae* members. Some of these natural plant products are phytoestrogens, indicating that their effects are similar to those of estrogen (Badgujar et al., 2014; Mercer et al., 2020), which can induce mammary epithelial cell (MECS) proliferation in lactating women and promote milk secretion (Setchell, 2001; Tsugami et al., 2017; Tsugami et al., 2020).

Milk generation is closely associated with serum estrogen, progesterone, and prolactin levels. Estrogen and progesterone stimulate mammary gland growth and development in pregnancy. Following delivery, the serum prolactin level increases, and this in turn substantially increases milk production. Thyroid hormone, insulin, low estrogen level, and progesterone promote pituitary prolactin secretion. In contrast, dopamine, high estrogen level, and progesterone inhibit pituitary prolactin secretion (Peña and Rosenfeld, 2001; Silva et al., 2020).

In various regions of the world, several herbs are used to promote lactation, with *T. foenum-graecum* L. and *F. vulgare* Mill being the most common (Forinash et al., 2012; Mortel and Mehta, 2013; Sim et al., 2013; Sim et al., 2014; Sim et al., 2015; Bazzano et al., 2016; Javan et al., 2017; Zheng et al., 2020). The origin of the two herbs resembles those indicated in the present study. *Trigonella foenum-graecum*, similar to *H. polybotrys* and *G. uralensis*, is a member of *Leguminosae*. Pharmacological studies have reported that phytoestrogens that are abundant in *T. foenum-graecum*, promote mammary gland growth, increase prolactin secretion, and stimulate milk production via antagonizing dopamine receptors (Foong et al., 2020). *Foeniculum vulgare*, similar to *A. sinensis* and *L. striatum*, is a member of *Apiaceae*. *Trans*-anethole in *F. vulgare* competes with dopamine for its receptors, blocks the inhibitory effect of dopamine on prolactin, and indirectly stimulates prolactin biosynthesis (Javan et al., 2017; Foong et al., 2020).

### Efficacy Analysis

TCM classifies medicinal materials according to their property and flavor. Their property include hot, warm, plain, cool, and cold. Hot and warm are opposites of cool and cold. Moreover, the degree of medicinal effects differs between warm and hot and between cool and cold materials (Zhang et al., 2020). Previous studies have reported that hot and warm traditional Chinese medicinal materials regulate the human endocrine system (Liang et al., 2013). Among the 19 medicinal materials identified in the galactogenous prescriptions collected here, 58% were warm. In general, warm medicinal materials have been widely used to increase milk secretion and their modes of action maybe associated with the endocrine system. The flavors of material medicines include sour, bitter, sweet, pungent, salty, plain, and astringent. Most sweet medicinal materials are tonics. TCM theory states that sweet medicinal materials are supplementing, moderating, and harmonizing (He et al., 2012). Here, sweet medicinal materials accounted for 54% of the 19 medicinal materials in the galactogenous prescriptions. These findings of the present study are consistent with the TCM theory.

### Analysis of the Core Medicinal Materials in the Prescriptions

*Angelica sinensis*, *H. polybotrys*, and *T. papyrifer* were the core components in the galactogenous prescriptions. *Angelica sinensis* has been prescribed to enrich the blood, whereas *H. polybotrys* has been administered to nourish the *qi* (Chang et al., 2020). Previous pharmacological studies on Chinese herbal medicines have reported that *A. sinensis*, *L. striatum*, *R. glutinosa*, and *Astragalus propinquus* Schischkin are used to treat anemia and enrich hemoglobin (Liang et al., 2004; Li et al., 2012; Jia et al., 2019; Liu J. et al., 2019). In the present study, all four medicinal materials were found to be frequently used. The secretion and nutritional value of milk are reduced in breastfeeding women with anemia (França et al., 2013). The mechanism by which galactogenous prescriptions enhance milk production may be associated with collaborative hemoglobin promotion by *A. sinensis*, *L. striatum*, *R. glutinosa*, and *A. propinquus*. As the flavor and function of *H. polybotrys* are similar to those of *A. propinquus*, the former is often used as a substitute for the latter and has become more popular in Taiwan (Lu et al., 2007). Most prescriptions obtained in this field investigation contained *H. polybotrys* (74%), whereas only 12% had *A. propinquus*, indicating that the application of *H. polybotrys* is common. *T. papyrifer* may dredge milk ducts. Approximately 77.3% of all lactating women with insufficient milk production used *T. papyrifer*; 25.6% of all users believed it could increase lactation and alleviate breast pain (Zheng et al., 2020). *T. papyrifer*mayinhibit inflammation and relieve breast pain associated with mastitis (Sugishita et al., 1983; Xu et al., 2016).

### Limitations and Future Works

This study had certain limitations that should be addressed in future research. The network diagram created with the TCMISS did not discriminate the frequency of application among medicinal materials. The demarcating line between pairs of medicinal materials only showed that they appeared more than 18 times. However, the frequency of application could not be further compared. Hence, a second chart must be plotted to better display the relationships among the medicinal materials examined here. Moreover, although numerous medicinal materials promoting lactation were collected in the present study, limited studies have explored or reported their modes of action. Furthermore, it remains unknown whether these materials induce adverse reactions in lactating women or their babies. Hence, the mechanisms underlying milk secretion/stimulation and the associated adverse effects of these medicinal materials merit further investigation. Furthermore, clinical trials are also needed to be verify their efficacies in future work.

## Conclusion

To the best of our knowledge, this study is the first ethnobotanical investigation of galactogenous prescriptions in Taiwan with the aim to assess the current status of TCM material prescribed and used to promote lactation. We generated valuable and comprehensive data on the galactogenous medicinal materials currently administered in Taiwan. The information compiled here will help preserve local knowledge regarding galactogenous medicinal materials in Taiwan and promote their prescription. Although galactogenous prescriptions have been used generally by Taiwanese lactating women, their function, efficacy, and safety warrant further investigation.

## Data Availability

The raw data supporting the conclusions of this article will be made available by the authors, without undue reservation, to any qualified researcher.
