# Supplementary material for: Ethnobotanical Survey of Natural Galactagogues Prescribed in Traditional Chinese Medicine Pharmacies in Taiwan
Source: Front Pharmacol. 2021 Feb 12;11:625869. doi: 10.3389/fphar.2020.625869 (PMC7928277; doi:10.3389/fphar.2020.625869)
Supplement: Supplementary file 1 [file DataSheet1.doc]

#

| **Table S1. Galactogenous medicinal materials in various regions of the world.** | | |
| --- | --- | --- |
|  | | |
| Region | Medicinal materials | Sources |
| America | *Trigonella foenum-graecum*, *Foeniculum vulgare*, *Galega officinalis* and *Silybum marianum* | (Bazzano et al., 2016; Forinash et al., 2012) |
| Australia | *Trigonella foenum-graecum*, *Zingiber officinale*, *Angelica sinensis*, *Matricaria chamomilla*, *Allium sativum*, *Cnicus benedictus*, *Vaccinium macrocarpon*, *Foeniculum vulgare*, *Aloe vera*, *Galega officinalis*, *Urtica dioica*, *Prunus spinosa*, *Asparagus racemosus* and *Mentha piperita* | (Sim et al., 2014, 2015; Sim et al., 2013) |
| China | *Tetrapanax papyrifer*, *Vaccaria segetalis*, *Trigonella foenum-graecum*,  *Foeniculum vulgare*, *Hordeum unlgare*, *Liquidambar formosana* and *Taraxacum officinale* | (Foong et al., 2020; Zheng et al., 2020) |
| India | *Curcuma longa* | (Medicine, 2006) |
| Italy | *Galega officinalis* | (Salatino et al., 2017) |
| Persia | *Foeniculum vulgare*, *Anethum graveolens*, *Pimpinella anisum*, *Nigella sativa* and *Vitex agnuscastus* | (Javan et al., 2017) |
| Republic of Vanuatu | *Colocasia esculenta*, *Ipomoea batatas* and *Carica papaya* | (Bourdy and Walter, 1992) |
| Thailand | *Musa* ×*paradisiac*, *Ocimum canum*, *Ocimum basilicum*, *Lagenaria siceraria*, *Zingiber officinale* and *Cucurbita pepo* | (Buntuchai et al., 2017; Paritakul et al., 2016) |
| Turkey | *Trigonella foenum-graecum* | (Mortel and Mehta, 2013) |

| **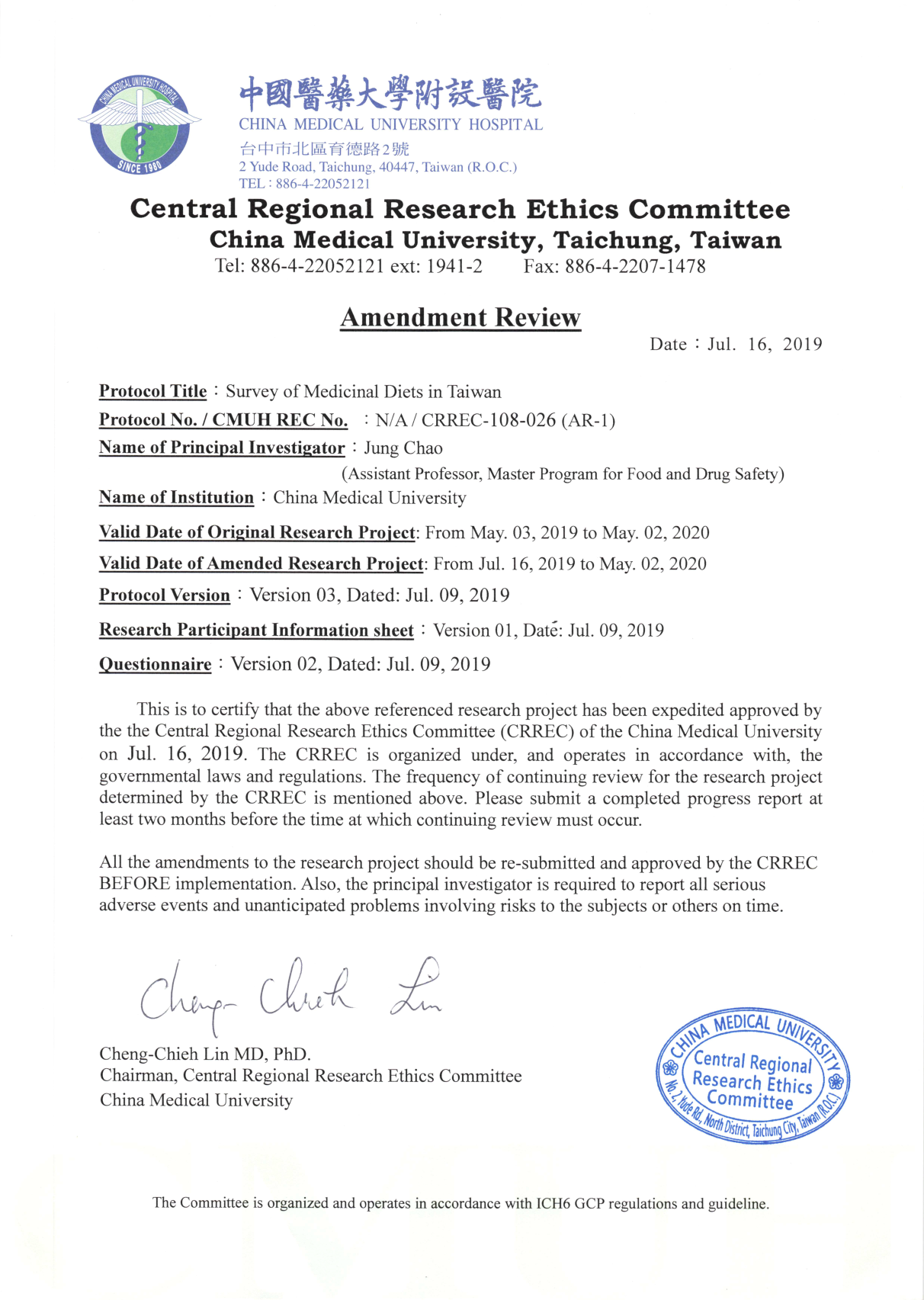** |
| --- |
| **Figure S1. Consent form from the Research Ethics Center of the Central Regional Research Ethics Committee of China Medical University (No. CRREC-108-026).** |

| 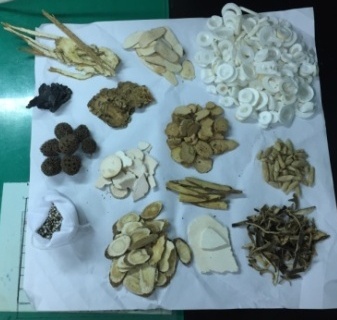 | 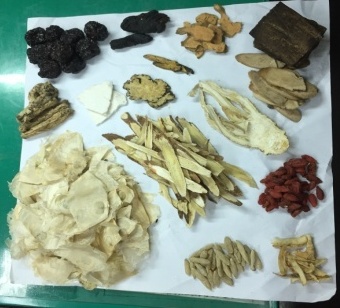 | 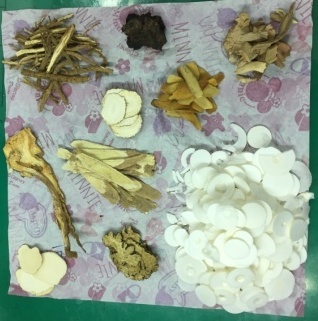 | 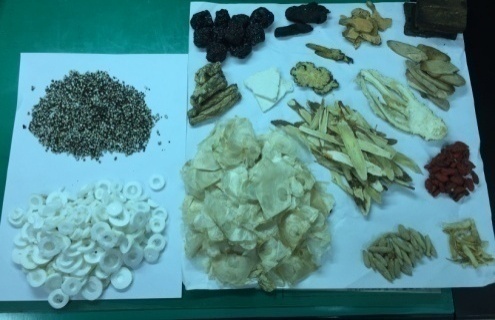 |
| --- | --- | --- | --- |
| 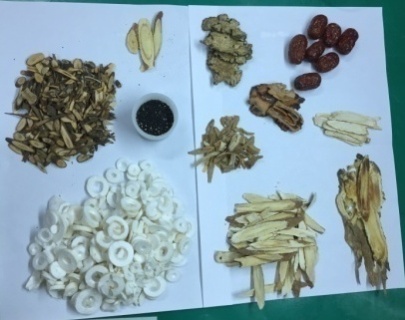 | 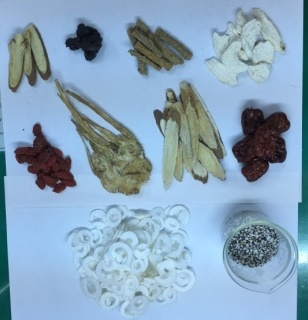 | 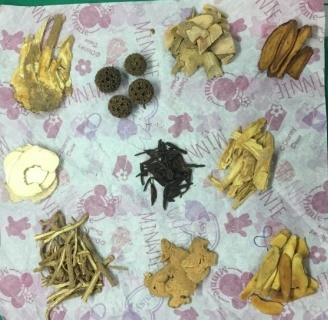 | 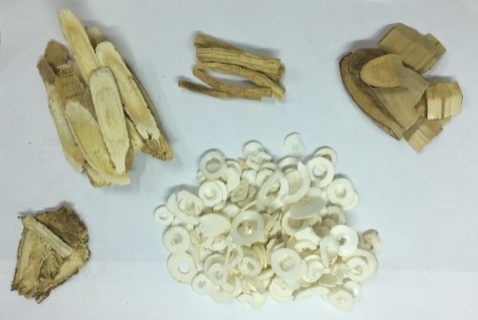 |
| 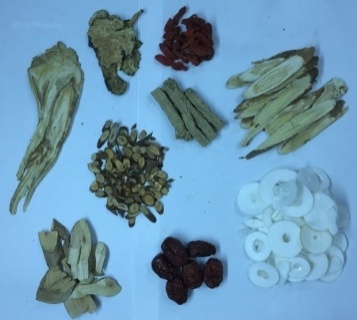 | 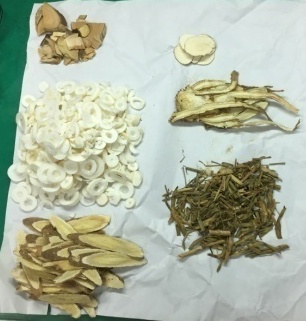 | 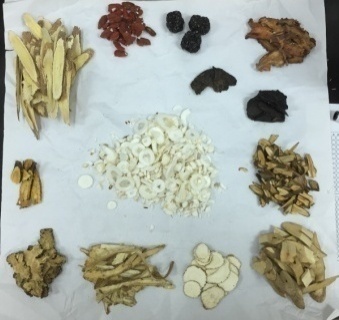 | 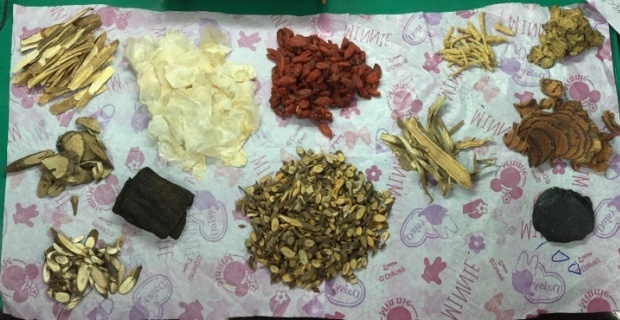 |
| 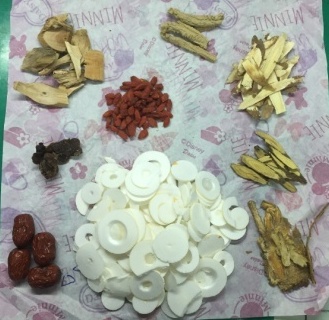 | 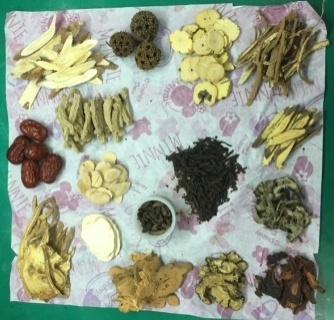 | 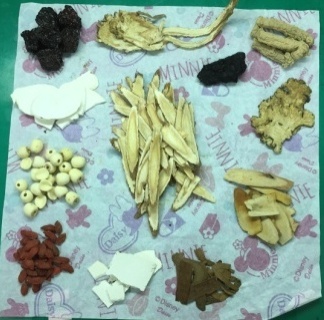 | 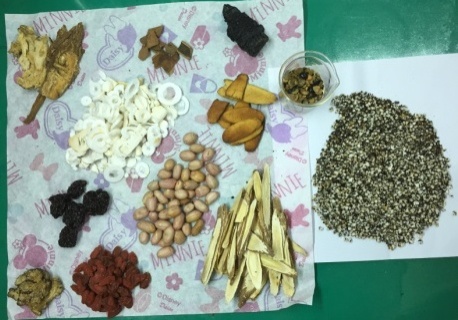 |
| 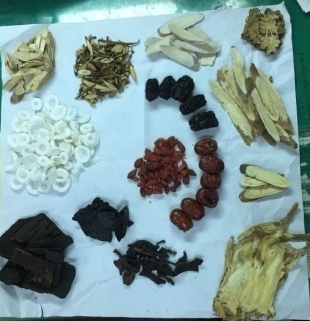 | 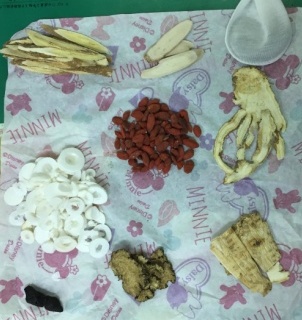 | 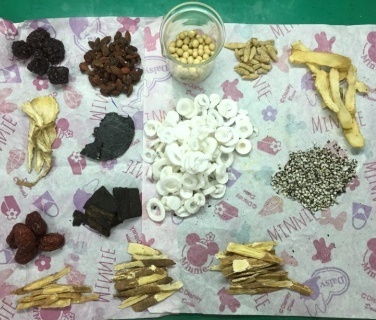 | 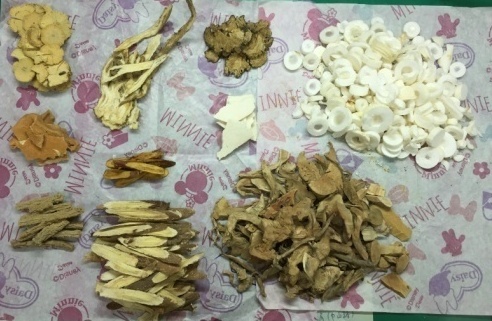 |
| 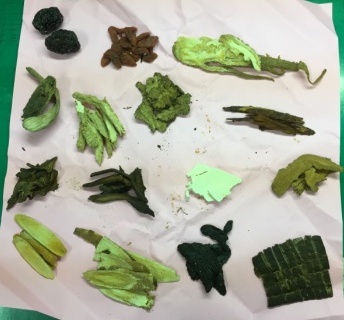 | 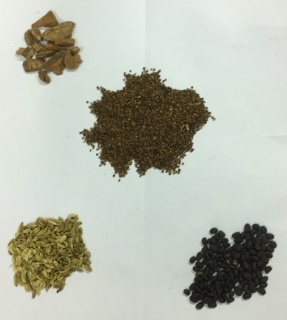 | 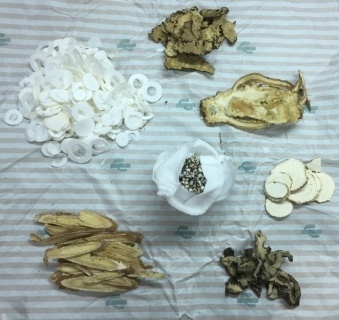 | 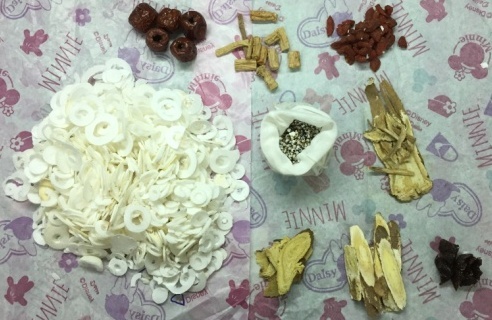 |
| 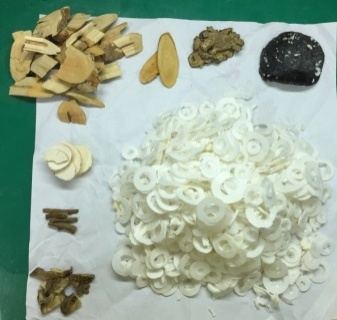 | 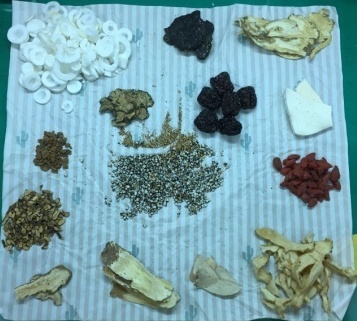 | 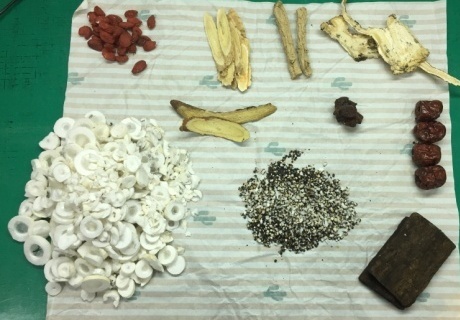 | 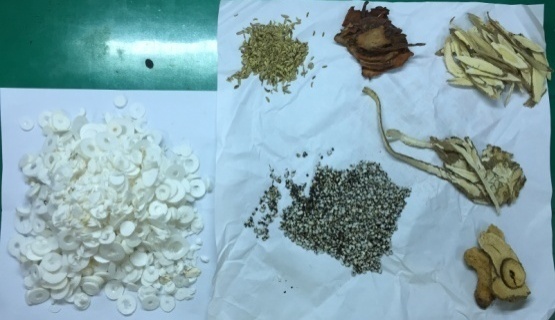 |
| 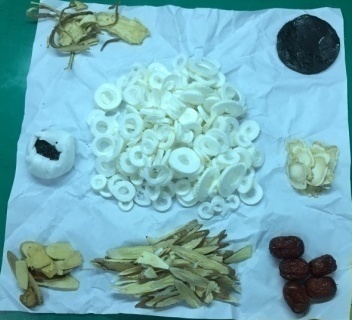 | 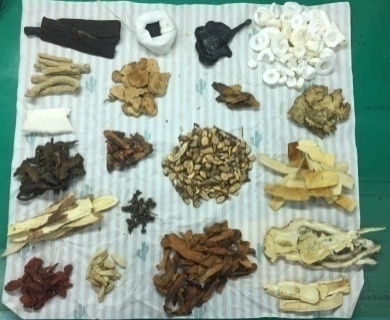 | 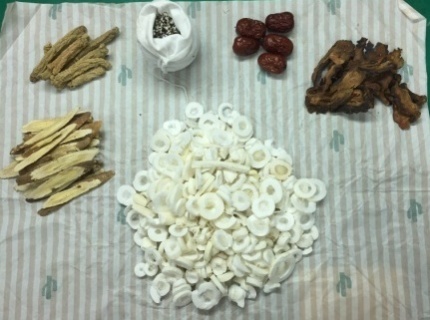 | 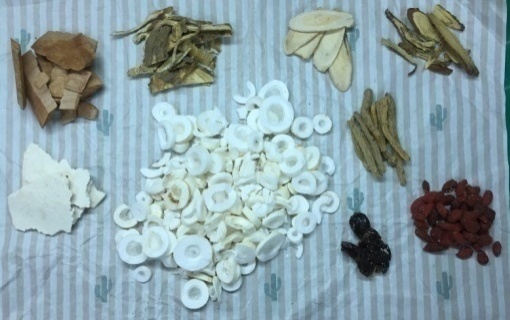 |
| 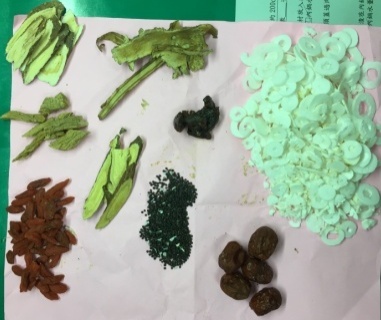 | 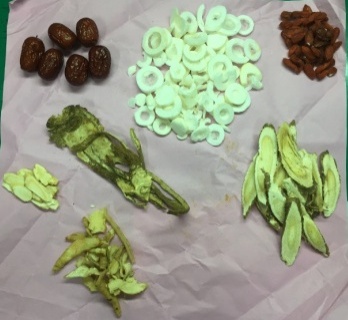 | 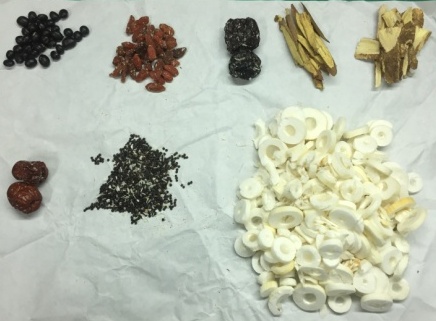 | 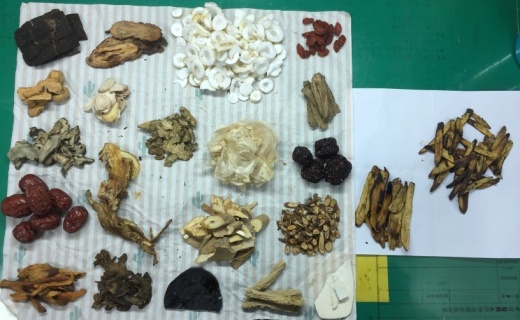 |
| 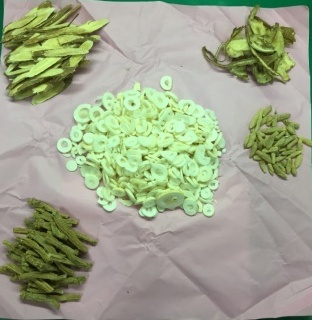 | 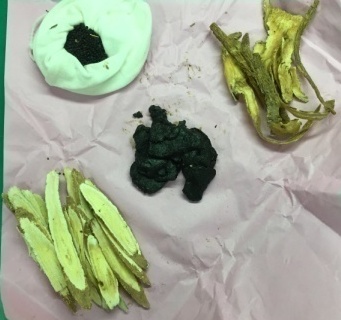 | 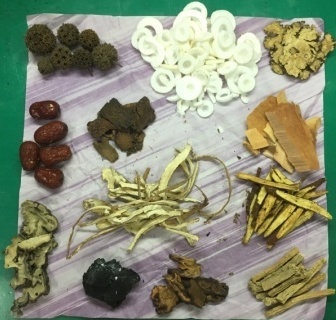 | 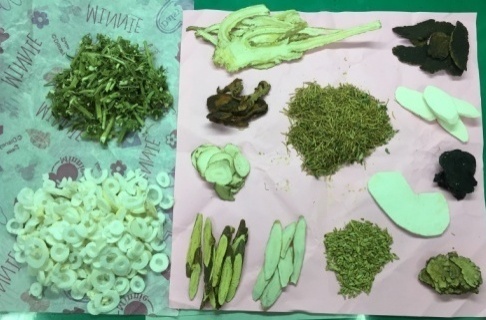 |
| 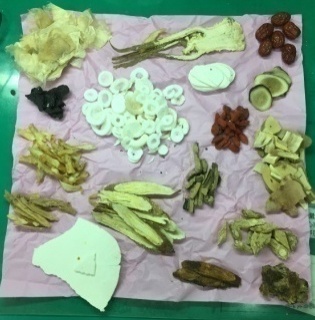 | 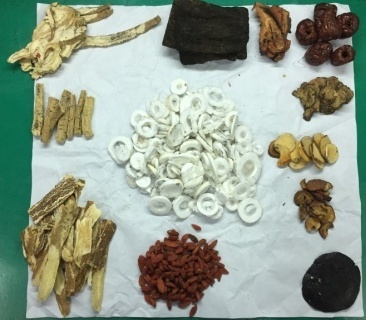 | 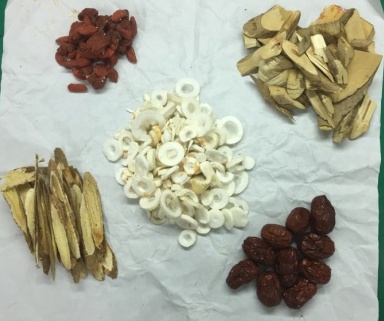 | 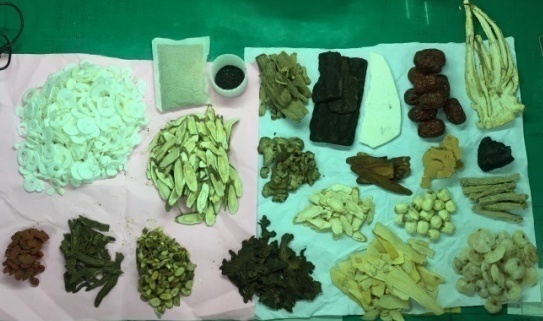 |
| 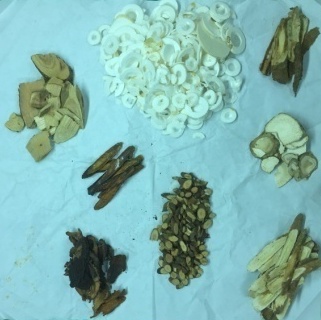 | 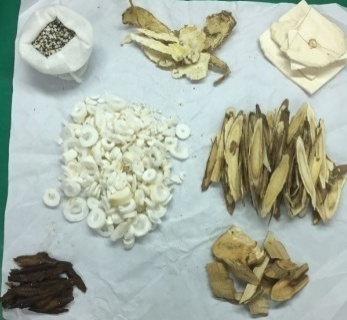 | 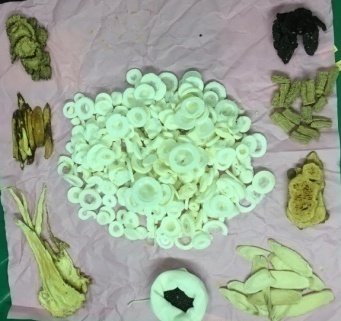 | 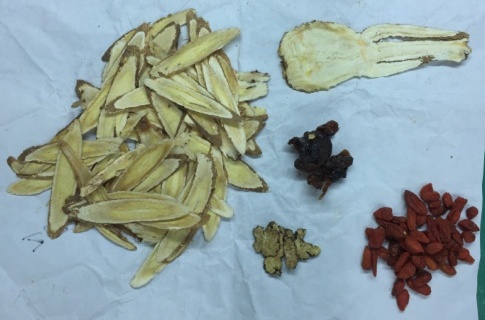 |
| 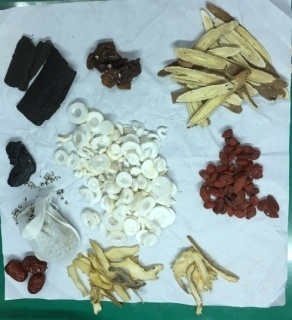 | 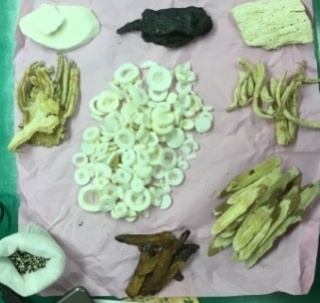 | 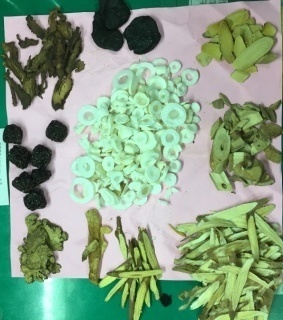 | 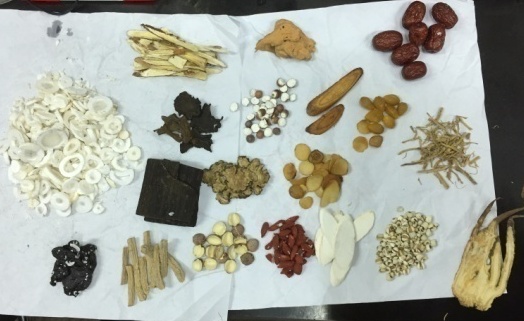 |
| 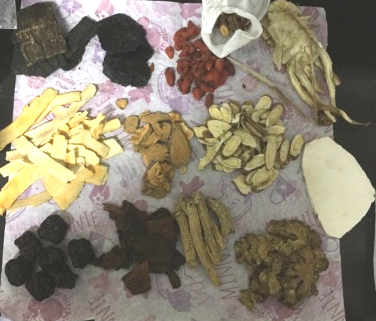 | 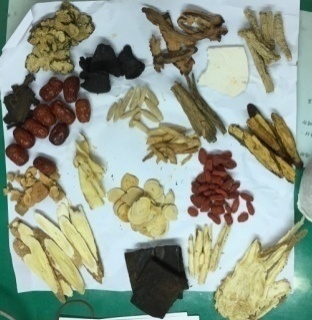 | 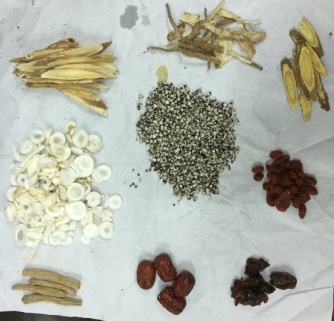 | 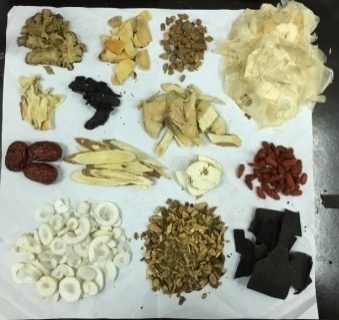 |
| 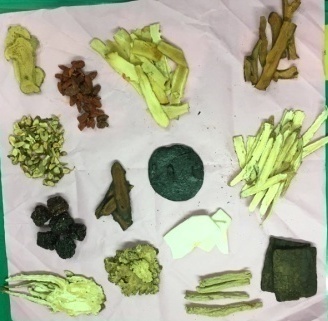 | 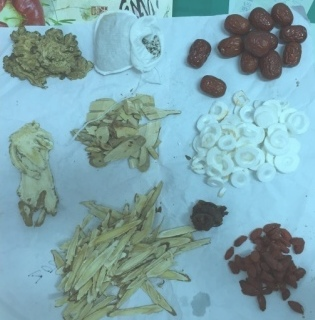 | 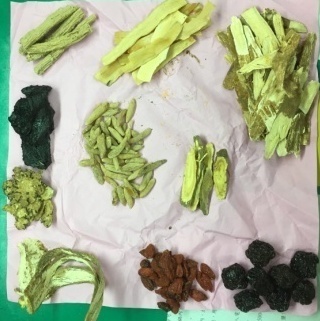 | 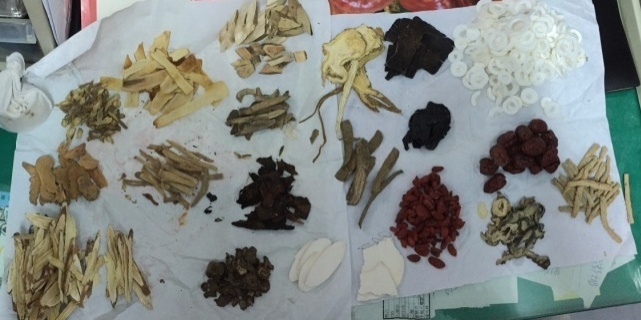 |
| 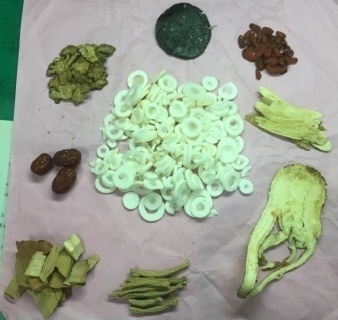 | 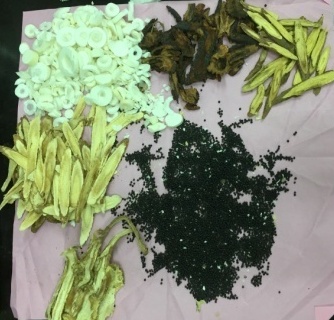 | 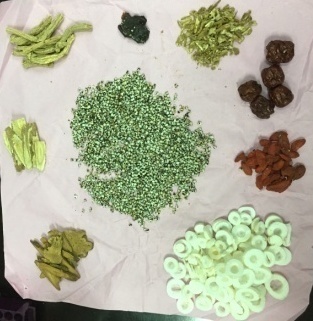 | 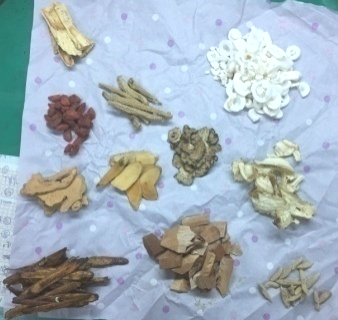 |
| 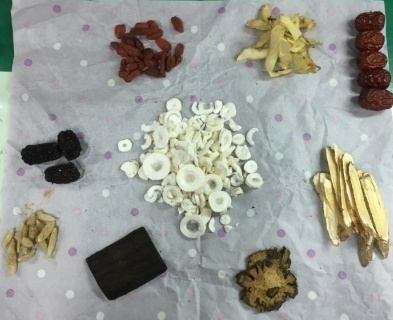 | 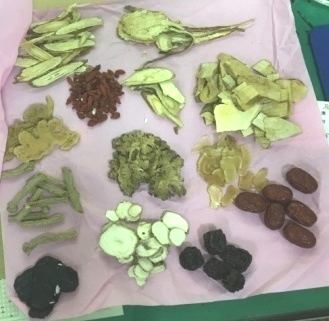 | 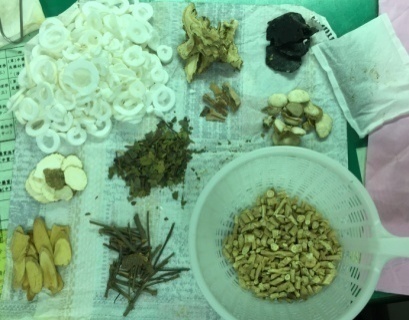 | 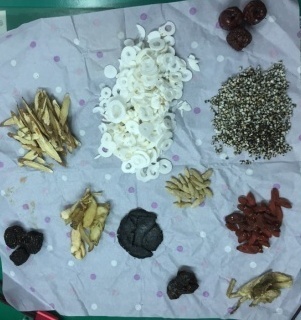 |
| 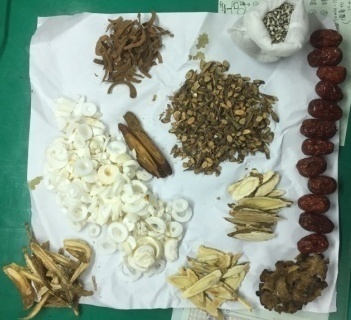 | 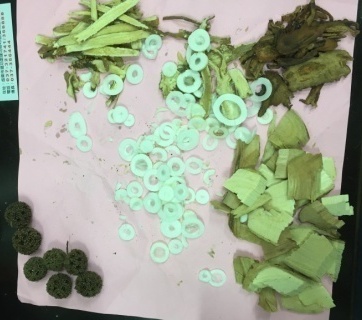 |  |  |
| Figure S2. Galactogenous prescriptions collected in this field investigation. | | | |

| Table S2. Characteristics of areaswhere TCM pharmacies located in. | | |
| --- | --- | --- |
|  | | |
| Code | Area | Description |
| YL1 | Yilan County, Taiwan | Oldest distribution center of traditional Chinese medicinal materials in eastern Taiwan |
| YL2 | Yilan County, Taiwan |
| NT1 | New Taipei City, Taiwan | Most populous city in Taiwan |
| NT2 | New Taipei City, Taiwan |
| NT3 | New Taipei City, Taiwan |
| NT4 | New Taipei City, Taiwan |
| NT5 | New Taipei City, Taiwan |
| NT6 | New Taipei City, Taiwan |
| TP1 | Taipei City, Taiwan | Dihua Streetin this city is a distribution center for TCM merchants |
| TP2 | Taipei City, Taiwan |
| TP3 | Taipei City, Taiwan |
| TP4 | Taipei City, Taiwan |
| TP5 | Taipei City, Taiwan |
| TP6 | Taipei City, Taiwan |
| TP7 | Taipei City, Taiwan |
| TP8 | Taipei City, Taiwan |
| TP9 | Taipei City, Taiwan |
| KL1 | Keelung City, Taiwan | Importation site of TCMs in Taiwan |
| KL2 | Keelung City, Taiwan |
| KL3 | Keelung City, Taiwan |
| KL4 | Keelung City, Taiwan |
| TY1 | Taoyuan City, Taiwan | A main *MesonaprocumbensHemsl* planting base in Taiwan |
| TY2 | Taoyuan City, Taiwan |
| TY3 | Taoyuan City, Taiwan |
| TY4 | Taoyuan City, Taiwan |
| TY5 | Taoyuan City, Taiwan |
| HC1 | Hsinchu City, Taiwan | A main *MesonaprocumbensHemsl* planting base in Taiwan |
| HC2 | Hsinchu City, Taiwan |
| HC3 | Hsinchu City, Taiwan |
| HC4 | Hsinchu City, Taiwan |
| ML1 | Miaoli County, Taiwan | Ethnic group consisting of Hakka people famous for their tea art. In recent years, they have planted *Chrysanthemum morifolium* and *Ziziphusjujuba* Mill. and their tea processing technology has been used to process traditional Chinese medicinal materials |
| ML2 | Miaoli County, Taiwan |
| TC1 | Taichung City, Taiwan | Local herbal medicine growing area |
| TC2 | Taichung City, Taiwan |
| TC3 | Taichung City, Taiwan |
| TC4 | Taichung City, Taiwan |
| TC5 | Taichung City, Taiwan |
| TC6 | Taichung City, Taiwan |
| TC7 | Taichung City, Taiwan |
| TC8 | Taichung City, Taiwan |
| CH1 | Changhua County, Taiwan | One of the oldest development zones and processing plant distribution centers in Taiwan |
| CH2 | Changhua County, Taiwan |
| CH3 | Changhua County, Taiwan |
| CH4 | Changhua County, Taiwan |
| CH5 | Changhua County, Taiwan |
| CH6 | Changhua County, Taiwan |
| CH7 | Changhua County, Taiwan |
| CH8 | Changhua County, Taiwan |
| CH9 | Changhua County, Taiwan |
| CH10 | Changhua County, Taiwan |
| CH11 | Changhua County, Taiwan |
| CH12 | Changhua County, Taiwan |
| CH13 | Changhua County, Taiwan |
| CH14 | Changhua County, Taiwan |
| NT1 | Taiwan Nantou County | Only city and county in Taiwan not bordering the sea.*Curcuma longa* and *Setariaviridis*are grown there |
| NT2 | Taiwan Nantou County |
| NT3 | Taiwan Nantou County |
| NT4 | Taiwan Nantou County |
| YL1 | Yunlin County, Taiwan | Main traditional Chinese medicinal material cultivation area of Taiwan. It propagates more than 100 species of medicinal materials |
| YL2 | Yunlin County, Taiwan |
| YL3 | Yunlin County, Taiwan |
| YL4 | Yunlin County, Taiwan |
| CY1 | Chiayi County, Taiwan | Large agricultural county planting *Taxilluschinensis* (DC.) Danser, *MesonaprocumbensHemsl*, *Houttuynia cordata*, and other medicinal materials |
| CY2 | Chiayi County, Taiwan |
| CY3 | Chiayi County, Taiwan |
| CY4 | Chiayi City, Taiwan |
| TN1 | Tainan City, Taiwan | Oldest reclaimed and developed area in Taiwan. It has several TCM pharmacies established more than 100 years ago |
| TN2 | Tainan City, Taiwan |
| TN3 | Tainan City, Taiwan |
| TN4 | Tainan City, Taiwan |
| TN5 | Tainan City, Taiwan |
| TN6 | Tainan City, Taiwan |
| TN7 | Tainan City, Taiwan |
| KH1 | Kaohsiung City, Taiwan | A distribution center for traditional Chinese medicinal material traders |
| KH2 | Kaohsiung City, Taiwan |
| KH3 | Kaohsiung City, Taiwan |
| KH4 | Kaohsiung City, Taiwan |
| KH5 | Kaohsiung City, Taiwan |
| KH6 | Kaohsiung City, Taiwan |
| KH7 | Kaohsiung City, Taiwan |
| KH8 | Kaohsiung City, Taiwan |
| KH9 | Kaohsiung City, Taiwan |
| KH10 | Kaohsiung City, Taiwan |
| KH11 | Kaohsiung City, Taiwan |
| KH12 | Kaohsiung City, Taiwan |
| PT1 | Pingtung County, Taiwan | A traditional Chinese medicinal material planting area in southern Taiwan. It produces dozens of traditional Chinese medicinal materials |
| PT2 | Pingtung County, Taiwan |
| HL1 | Hualien County, Taiwan | A traditional Chinese medicinal material planting area in eastern Taiwan. It grows*Chrysanthemum morifolium*, *Hibiscus sabdariffa*, *Hemerocallis citrina,* and *Angelicasinensis*(Oliv.) Diels |
| TT1 | Taitung County, Taiwan | A traditional Chinese medicinal material planting area in eastern Taiwan. It generates*Chrysanthemum morifolium*, *Hibiscus sabdariffa*, *Hemerocallis citrina,* and *Angelicasinensis*(Oliv.) Dielss |
| TT2 | Taitung County, Taiwan |

| **Table S3. Properties of 81 medicinal materials in galactogenous prescriptions and their pharmacopeia inclusion status** | | | | | | | | | | | | | |
| --- | --- | --- | --- | --- | --- | --- | --- | --- | --- | --- | --- | --- | --- |
|  | | | | | | | | | | | | | |
| **No** | **Scientific name** | **Code** | **Kingdom** | **Family** | **Part of use** | **Local name** | **RFC** | **Additional information** | | | | |  |
|  |  |  |  |  |  |  |  | **Taiwan Herbal Pharmacopeia 3rd Edition** | **Pharmacopoeia of the People’s Republic of China 2020 Edition** | | **Chinese Materia Medica** | |  |
| **1** | *Angelica sinensis*  (Oliv.) Diels | AS | Plantae | Apiaceae | Radix | Tang kuei  當歸 | 0.93 | Y | | Y | | Y |  |
| **2** | *Tetrapanax* *papyrifer*  (Hook.) K. Koch | TP | Plantae | Araliaceae | Medulla | T’ungts’ao  通草 | 0.86 | Y | | Y | | Y |  |
| **3** | *Hedysarum* *polybotrys* Hand.-Mazz. | HP | Plantae | Leguminosae | Radix | Hung ch’i  紅耆 | 0.83 | Y | | Y | | Y |  |
| **4** | *Lycium* *chinense* Mill. | LCh | Plantae | Solanaceae | Fructus | Kou ch’i  枸杞 | 0.64 | Y | | Y | | Y |  |
| **5** | *Glycyrrhiza* *uralensis* Fisch. | GU | Plantae | Leguminosae | Radix | Kan ts’ao  甘草 | 0.56 | Y | | Y | | Y |  |
| **6** | *Ligusticum* *striatum*  DC. | LS | Plantae | Apiaceae | Rhizoma | Ch’uanch’iung  川芎 | 0.54 | Y | | Y | | Y |  |
| **7** | *Ziziphus* *jujuba* Mill. | ZJR | Plantae | Rhamnaceae | Fructus | Hung tsao  紅棗 | 0.51 | Y | | Y | | Y |  |
| **8** | *Vaccaria* *hispanica*  (Mill.) Rauschert | VH | Plantae | Caryophyllaceae | Semen | Wang puliuhsing  王不留行 | 0.49 | Y | | Y | | Y |  |
| **9** | *Codonopsis* *pilosula*  (Franch.) Nannf. | CPil | Plantae | Campanulaceae | Radix | Tang san  黨參 | 0.49 | Y | | Y | | Y |  |
| **10** | *Rehmannia* *glutinosa*  (Gaertn.) DC. | RGC | Plantae | Plantaginaceae | Radix | Shu tihuang  熟地黃 | 0.47 |  | | Y | | Y |  |
| **11** | *Paeonia* *lactiflora*  Pall. | PLW | Plantae | Paeoniaceae | Radix | Pai shao  白芍 | 0.39 | Y | | Y | | Y |  |
| **12** | *Melastoma* *malabathricum* L. | MM | Plantae | Melastomataceae | Caulis & Radix | Yeh mu tan  野牡丹 | 0.38 | N | | N | | Y |  |
| **13** | *Atractylodes* *macrocephala* Koidz. | AM | Plantae | Compositae | Rhizoma | Pai chu  白朮 | 0.26 | Y | | Y | | Y |  |
| **14** | *Chaenomeles* *speciosa*  (Sweet) Nakai | CS | Plantae | Rosaceae | Fructus | Mu kua  木瓜 | 0.23 | Y | | Y | | Y |  |
| **15** | *Cinnamomum* *cassia* (L.) J. Presl | CCR | Plantae | Lauraceae | Ramulus | Kueichih  桂枝 | 0.23 | Y | | Y | | Y |  |
| **16** | *Eucommia* *ulmoides* Oliv. | EU | Plantae | Eucommiaceae | Cortex | Tu chung  杜仲 | 0.22 | Y | | Y | | Y |  |
| **17** | *Poriacocos*(Schwein.) F.A. Wolf | PCo | Fungi | Polyporaceae | Sclerotia | Fu ling  茯苓 | 0.21 | Y | | Y | | Y |  |
| **18** | *Ziziphus* *jujuba* Mill. | ZJB | Plantae | Rhamnaceae | Fructus | Heitsao  黑棗 | 0.20 | Y | | Y | | Y |  |
| **19** | *Dimocarpus* *longan* Lour. | DL | Plantae | Sapindaceae | Arillus | Kueiyüan  桂圓 | 0.20 | N | | N | | Y |  |
| **20** | *Astragalus* *propinquus* Schischkin | AP | Plantae | Leguminosae | Radix | Huang ch’i  黃耆 | 0.12 | Y | | Y | | Y |  |
| **21** | *Cinnamomum* *cassia* (L.) J. Presl | CCC | Plantae | Lauraceae | Cortex | Joukuei  肉桂 | 0.12 | Y | | Y | | Y |  |
| **22** | *Dioscorea* *oppositifolia* L. | DO | Plantae | Dioscoreaceae | Rhizoma | Shan yao  山藥 | 0.12 | Y | | Y | | Y |  |
| **23** | *Ophiopogon* *japonicus* (Thunb.) Ker Gawl. | OJ | Plantae | Asparagaceae | Radix | Mai mên tung  麥門冬 | 0.12 | Y | | Y | | Y |  |
| **24** | *Angelica* *dahurica* (Hoffm.) Benth. &Hook.f. ex Franch. & Sav. | Ada | Plantae | Apiaceae | Radix | Pai chih  白芷 | 0.11 | Y | | Y | | Y |  |
| **25** | *Polygonatum* *odoratum*  (Mill.) Druce | PO | Plantae | Asparagaceae | Rhizoma | Yü chu  玉竹 | 0.11 | Y | | Y | | Y |  |
| **26** | *Trichosanthes* *kirilowii*  Maxim. | TK | Plantae | Cucurbitaceae | Radix | Kualoukên  栝樓根 | 0.10 | Y | | Y | | Y |  |
| **27** | *Oroxylum* *indicum* (L.) Kurz | OI | Plantae | Bignoniaceae | Semen | Mu hu tieh  木蝴蝶 | 0.08 | Y | | Y | | Y |  |
| **28** | *Cibotium* *barometz* (L.) J.Sm. | CB | Plantae | Cibotiaceae | Rhizoma | Kou chi  狗脊 | 0.07 | Y | | Y | | Y |  |
| **29** | *Clematis* *montana*  Buch.-Ham. ex DC. | CM | Plantae | Ranunculaceae | Caulis | Ch’uan mu t’ung  川木通 | 0.07 | Y | | Y | | Y |  |
| **30** | *Foeniculum* *vulgare* Mill. | FV | Plantae | Apiaceae | Fructus | Hsiao hui hsiang  小茴香 | 0.07 | Y | | Y | | Y |  |
| **31** | *Panax* *quinquefolius* L. | PQ | Plantae | Araliaceae | Radix | Hsi yang shên  西洋參 | 0.07 | Y | | Y | | Y |  |
| **32** | *Platycodon* *grandiflorus*  (Jacq.) A. DC. | PGr | Plantae | Campanulaceae | Radix | Chiehkêng  桔梗 | 0.07 | Y | | Y | | Y |  |
| **33** | *Strobilanthes* *forrestii* Diels | SF | Plantae | Acanthaceae | Radix | Wei niuhsi  味牛膝 | 0.07 | N | | N | | Y |  |
| **34** | *Citrus* *reticulata* Blanco | CRe | Plantae | Rutaceae | Pericarpium | Ch’ênp’i  陳皮 | 0.06 | Y | | Y | | Y |  |
| **35** | *Dipsacus* *inermis* Wall. | DI | Plantae | Caprifoliaceae | Radix | Hsü tuan  續斷 | 0.06 | Y | | Y | | Y |  |
| **36** | *Liquidambar* *formosana*  Hance | LF | Plantae | Altingiaceae | Fructus | Lu lut’ung  路路通 | 0.06 | Y | | Y | | Y |  |
| **37** | *Achyranthes* *bidentata*  Blume | AB | Plantae | Amaranthaceae | Radix | Huainiuhsi  懷牛膝 | 0.04 | Y | | Y | | Y |  |
| **38** | *Nelumbo* *nucifera* Gaertn. | NNSe | Plantae | Nelumbonaceae | Semen | Lien tzu  蓮子 | 0.04 | Y | | Y | | Y |  |
| **39** | *Araiostegia* *divaricata*  (Blume) M. Kato | Adi | Plantae | Davalliaceae | Rhizoma | Ta yeh ku sui pu  大葉骨碎補 | 0.03 | N | | N | | Y |  |
| **40** | *Bupleurum* *chinense* DC. | BC | Plantae | Apiaceae | Radix | Ch’ai hu  柴胡 | 0.03 | Y | | Y | | Y |  |
| **41** | *Glycine* *max* (L.) Merr. | GMB | Plantae | Leguminosae | Semen | Hei ta tou  黑大豆 | 0.03 | Y | | Y | | Y |  |
| **42** | *Morinda* *officinalis* F.C.How | MO | Plantae | Rubiaceae | Radix | Pa chi t’ien  巴戟天 | 0.03 | Y | | Y | | Y |  |
| **43** | *Pueraria* *montana* var.  *lobata* (Willd.) Sanjappa& Pradeep | PM | Plantae | Leguminosae | Radix | Kêkên  葛根 | 0.03 | Y | | Y | | Y |  |
| **44** | *Cervus nippon*Temminck | CNC | Animalia | Cervidae | Cornupantotrichum | Lu jung  鹿茸 | 0.02 | N | | Y | | Y |  |
| **45** | *Cuscuta* *australis* R.Br. | Cau | Plantae | Convolvulaceae | Semen | T’ussu tzu  菟絲子 | 0.02 | Y | | Y | | Y |  |
| **46** | *Polygonatum* *cyrtonema*  Hua | PCy | Plantae | Asparagaceae | Rhizoma | Huang ching  黃精 | 0.02 | Y | | Y | | Y |  |
| **47** | *Reynoutria* *multiflora*  (Thunb.) Moldenke | RM | Plantae | Polygonaceae | Radix | Hê shou wu  何首烏 | 0.02 | Y | | Y | | Y |  |
| **48** | *Salvia* *miltiorrhiza* Bunge | SM | Plantae | Lamiaceae | Radix et Rhizoma | Tan shên  丹參 | 0.02 | Y | | Y | | Y |  |
| **49** | *Saposhnikovia* *divaricata*  (Turcz.) Schischk. | SD | Plantae | Apiaceae | Radix | Fang fêng  防風 | 0.02 | Y | | Y | | Y |  |
| **50** | *Spatholobus* *suberectus*  Dunn | SS | Plantae | Leguminosae | Caulis | Chi hsieht’êng  雞血藤 | 0.02 | Y | | Y | | Y |  |
| **51** | *Akebia* *quinata* (Houtt.) Decne. | AQ | Plantae | Lardizabalaceae | Caulis | Wu yeh mu t’ung  五葉木通 | 0.01 | Y | | Y | | Y |  |
| **52** | *Arachis* *hypogaea* L. | AH | Plantae | Leguminosae | Semen | Lo huashêng  落花生 | 0.01 | N | | N | | Y |  |
| **53** | *Carthamus* *arborescens* L. | CAr | Plantae | Compositae | Flos | Hung hua  紅花 | 0.01 | Y | | Y | | Y |  |
| **54** | *Cervus nippon*Temminck | CNG | Animalia | Cervidae | Genitalia | Lu pien  鹿鞭 | 0.01 | N | | N | | Y |  |
| **55** | *Cinnamomum* *cassia* (L.) J. Presl | CCF | Plantae | Lauraceae | Fructus | Kuei ting  桂丁 | 0.01 | N | | N | | Y |  |
| **56** | *Cistanche* *deserticola*  Y.C. Ma | CD | Plantae | Orobanchaceae | Herba | Jouts’ungjung  肉蓯蓉 | 0.01 | Y | | Y | | Y |  |
| **57** | *Coix* *lacryma-jobi* L. | CL | Plantae | Poaceae | Semen | I ijên  薏苡仁 | 0.01 | Y | | Y | | Y |  |
| **58** | *Crataegus* *pinnatifida*  Bunge | Cpin | Plantae | Rosaceae | Fructus | Shan cha  山楂 | 0.01 | Y | | Y | | Y |  |
| **59** | *Cullen* *corylifolium* (L.) Medik. | CCo | Plantae | Leguminosae | Fructus | Pu kuchih  補骨脂 | 0.01 | Y | | Y | | Y |  |
| **60** | *Cyathula* *officinalis* K.C.Kuan | CO | Plantae | Amaranthaceae | Radix | Ch’uanniuhsi  川牛膝 | 0.01 | Y | | Y | | Y |  |
| **61** | *Cyperus* *rotundus* L. | CRo | Plantae | Cyperaceae | Rhizoma | Hsiang fu  香附 | 0.01 | Y | | Y | | Y |  |
| **62** | *Dianthus* *superbus* L. | DS | Plantae | Caryophyllaceae | Herba | Ch’ümai  瞿麥 | 0.01 | Y | | Y | | Y |  |
| **63** | *Euryale* *ferox* Salisb. | EF | Plantae | Nymphaeaceae | Semen | Ch’ienshih  芡實 | 0.01 | Y | | Y | | Y |  |
| **64** | *Flemingia* *prostrata* Roxb. | FP | Plantae | Leguminosae | Radix | Manhsingch’ienchin pa  蔓性千斤拔 | 0.01 | N | | N | | Y |  |
| **65** | *Fritillaria* *thunbergii* Miq. | FT | Plantae | Liliaceae | Bulbus | Chêpei mu  浙貝母 | 0.01 | Y | | Y | | Y |  |
| **66** | *Gastrodia* *elata* Blume | GE | Plantae | Orchidaceae | Rhizoma | T’ien ma  天麻 | 0.01 | Y | | Y | | Y |  |
| **67** | *Ginkgo* *biloba* L. | GB | Plantae | Ginkgoaceae | Semen | Pai kuo  白果 | 0.01 | Y | | Y | | Y |  |
| **68** | *Glycine* *max* (L.) Merr. | GMY | Plantae | Leguminosae | Semen | Huang ta tou  黃大豆 | 0.01 | N | | N | | Y |  |
| **69** | *Leonurus* *japonicus* Houtt. | LJ | Plantae | Lamiaceae | Herba | I mu ts’ao  益母草 | 0.01 | Y | | Y | | Y |  |
| **70** | *Luffa* *cylindrica* (L.) M. Roem. | LCy | Plantae | Cucurbitaceae | Fructus retinervus | Ssukua lao  絲瓜絡 | 0.01 | N | | Y | | Y |  |
| **71** | *Manihot* *esculenta* Crantz | ME | Plantae | Euphorbiaceae | Radix | Mu shu  木薯 | 0.01 | N | | N | | Y |  |
| **72** | *Nelumbo* *nucifera* Gaertn. | NNSt | Plantae | Nelumbonaceae | Stamen | Lien hsü  蓮鬚 | 0.01 | Y | | Y | | Y |  |
| **73** | *Paeonia* *lactiflora* Pall. | PLR | Plantae | Paeoniaceae | Radix | Ch’ih shao  赤芍 | 0.01 | Y | | Y | | Y |  |
| **74** | *Panax* *ginseng* C.A.Mey. | PGi | Plantae | Araliaceae | Radix | Jênshênhsü  人參鬚 | 0.01 | Y | | Y | | Y |  |
| **75** | *Piper* *nigrum* L. | PN | Plantae | Piperaceae | Fructus | Pai hu chiao  白胡椒 | 0.01 | Y | | Y | | Y |  |
| **76** | *Prunus* *persica* (L.) Batsch | PP | Plantae | Rosaceae | Semen | T’aojên  桃仁 | 0.01 | Y | | Y | | Y |  |
| **77** | *Rehmannia* *glutinosa*  (Gaertn.) DC. | RGR | Plantae | Plantaginaceae | Radix | Shêngtihuang  生地黃 | 0.01 | Y | | Y | | Y |  |
| **78** | *Scapharcainflata*(Reeve) | SInf | Animalia | Arcidae | Concha | Walêng tzu  瓦楞子 | 0.01 | N | | N | | Y |  |
| **79** | *Scrophularia* *ningpoensis*  Hemsl. | SN | Plantae | Scrophulariaceae | Radix | Hsüanshên  玄參 | 0.01 | Y | | Y | | Y |  |
| **80** | *Sesamum* *indicum* L. | SInd | Plantae | Pedaliaceae | Semen | Hu ma jên  胡麻仁 | 0.01 | Y | | Y | | Y |  |
| **81** | *Taxillus* *chinensis* (DC.) Danser | TC | Plantae | Loranthaceae | Ramulus | Sang chi shêng  桑寄生 | 0.01 | Y | | Y | | Y |  |

| **Table S4. Frequently occurring drug pairs in galactogenous prescriptions (frequency ≥41; RFC ≥ 0.45)** | | |
| --- | --- | --- |
| No. | Drug combination | Frequency |
| 1 | *Tetrapanaxpapyrifer, Angelica sinensis* | 71 |
| 2 | *Angelica sinensis, Hedysarumpolybotrys* | 69 |
| 3 | *Tetrapanaxpapyrifer, Hedysarumpolybotrys* | 63 |
| 4 | *Angelica sinensis, Lyciumchinense* | 54 |
| 5 | *Lyciumchinense, Hedysarumpolybotrys* | 54 |
| 6 | *Tetrapanaxpapyrifer, Lyciumchinense* | 48 |
| 7 | *Glycyrrhiza uralensis, Angelica sinensis* | 48 |
| 8 | *Ligusticum striatum, Angelica sinensis* | 47 |
| 9 | *Ziziphus jujuba, Hedysarumpolybotrys* | 44 |
| 10 | *Tetrapanaxpapyrifer, Vaccariahispanica* | 43 |
| 11 | *Tetrapanaxpapyrifer, Glycyrrhiza uralensis* | 43 |
| 12 | *Glycyrrhiza uralensis, Hedysarumpolybotrys* | 43 |
| 13 | *Codonopsispilosula, Angelica sinensis* | 43 |
| 14 | *Tetrapanaxpapyrifer, Ziziphus jujuba* | 42 |
| 15 | *Ligusticum striatum, Hedysarumpolybotrys* | 42 |
| 16 | *Rehmannia* *glutinosa, Angelica sinensis* | 41 |
| 17 | *Vaccariahispanica, Angelica sinensis* | 41 |
| 18 | *Ziziphus jujuba, Angelica sinensis* | 41 |
| *RFC, relative frequency of citation. | | |

| **Table S5.** | |  |
| --- | --- | --- |
| Analysis of correlation rules of galactogenous prescriptions (Confidence score = 1) | |  |
| No | Combinational correlations ofrawmaterials | |
| 1 | *Ligusticum striatum, Paeonia lactiflora, Lyciumchinense* → *Angelica sinensis* | |
| 2 | *Ligusticum striatum, Paeonia lactiflora, Lyciumchinense, Hedysarumpolybotrys* → *Angelica sinensis* | |
| 3 | *Ligusticum striatum, Cinnamomum cassia* → *Hedysarumpolybotrys* | |
| 4 | *Ligusticum striatum, Cinnamomum cassia* → *Angelica sinensis* | |
| 5 | *Ligusticum striatum, Cinnamomum cassia* → *Angelica sinensis, Hedysarumpolybotrys* | |
| 6 | *Ligusticum striatum, Cinnamomum cassia, Hedysarumpolybotrys → Angelica sinensis* | |
| 7 | *Ligusticum striatum, Angelica sinensis, Cinnamomum cassia → Hedysarumpolybotrys* | |
| 8 | *Ligusticum striatum, Codonopsispilosula → Angelica sinensis* | |
| 9 | *Ligusticum striatum, Codonopsispilosula, Glycyrrhiza uralensis → Angelica sinensis* | |
| 10 | *Ligusticum striatum, Codonopsispilosula, Lyciumchinense → Angelica sinensis* | |
| 11 | *Ligusticum striatum, Codonopsispilosula, Lyciumchinense, Hedysarumpolybotrys → Angelica sinensis* | |
| 12 | *Ligusticum striatum, Codonopsispilosula, Hedysarumpolybotrys → Angelica sinensis* | |
| 13 | *Vaccariahispanica, Ligusticum striatum → Tetrapanaxpapyrifer* | |
| 14 | *Vaccariahispanica, Ligusticum striatum → Tetrapanaxpapyrifer, Angelica sinensis* | |
| 15 | *Vaccariahispanica, Ligusticum striatum → Angelica sinensis* | |
| 16 | *Vaccariahispanica, Ligusticum striatum, Hedysarumpolybotrys → Tetrapanaxpapyrifer* | |
| 17 | *Vaccariahispanica, Ligusticum striatum, Hedysarumpolybotrys → Tetrapanaxpapyrifer, Angelica sinensis* | |
| 18 | *Vaccariahispanica, Ligusticum striatum, Hedysarumpolybotrys → Angelica sinensis* | |
| 19 | *Vaccariahispanica, Ligusticum striatum, Angelica sinensis → Tetrapanaxpapyrifer* | |
| 20 | *Vaccariahispanica, Ligusticum striatum, Angelica sinensis, Hedysarumpolybotrys → Tetrapanaxpapyrifer* | |
| 21 | *Vaccariahispanica, Glycyrrhiza uralensis* → *Tetrapanaxpapyrifer* | |
| 22 | *Vaccariahispanica, Glycyrrhiza uralensis, Hedysarumpolybotrys* → *Tetrapanaxpapyrifer* | |
| 23 | *Vaccariahispanica, Glycyrrhiza uralensis, Angelica sinensis* → *Tetrapanaxpapyrifer* | |
| 24 | *Vaccariahispanica, Glycyrrhiza uralensis, Angelica sinensis, Hedysarumpolybotrys* → *Tetrapanaxpapyrifer* | |
| 25 | *Vaccariahispanica, Lyciumchinense* → *Tetrapanaxpapyrifer* | |
| 26 | *Vaccariahispanica, Lyciumchinense, Hedysarumpolybotrys* → *Tetrapanaxpapyrifer* | |
| 27 | *Vaccariahispanica, Ziziphus jujuba* → *Tetrapanaxpapyrifer* | |
| 28 | *Vaccariahispanica, Ziziphus jujuba, Angelica sinensis* → *Tetrapanaxpapyrifer* | |
| 29 | *Vaccariahispanica, Ziziphus jujuba, Lyciumchinense* → *Tetrapanaxpapyrifer* | |
| 30 | *Vaccariahispanica, Ziziphus jujuba, Hedysarumpolybotrys* → *Tetrapanaxpapyrifer* | |
| 31 | *Vaccariahispanica, Ziziphus jujuba, Angelica sinensis, Hedysarumpolybotrys* → *Tetrapanaxpapyrifer* | |
| 32 | *Vaccariahispanica, Angelica sinensis, Lyciumchinense* → *Tetrapanaxpapyrifer* | |
| 33 | *Vaccariahispanica, Angelica sinensis, Lyciumchinense, Hedysarumpolybotrys* → *Tetrapanaxpapyrifer* | |
| 34 | *AtractylodesmacrocephalaKoidz.* → *Angelica sinensis* | |
| 35 | *AtractylodesmacrocephalaKoidz., Hedysarumpolybotrys* → *Angelica sinensis* | |
| 36 | *Paeonia lactiflora, Lyciumchinense* → *Angelica sinensis* | |
| 37 | *Paeonia lactiflora, Lyciumchinense, Hedysarumpolybotrys* → *Angelica sinensis* | |
| 38 | *Eucommia ulmoides* → *Lyciumchinense* | |
| 39 | *Eucommia ulmoides, Hedysarumpolybotrys* → *Lyciumchinense* | |
| 40 | *Eucommia ulmoides, Angelica sinensis* → *Lyciumchinense* | |
| 41 | *Cinnamomum cassia* → *Hedysarumpolybotrys* | |
| 42 | *Cinnamomum cassia* → *Angelica sinensis* | |
| 43 | *Cinnamomum cassia* → *Angelica sinensis, Hedysarumpolybotrys* | |
| 44 | *Cinnamomum cassia, Hedysarumpolybotrys* → *Angelica sinensis* | |
| 45 | *Poriacocos* → *Angelica sinensis* | |
| 46 | *Tetrapanaxpapyrifer, Vaccariahispanica, Ligusticum striatum* → *Angelica sinensis* | |
| 47 | *Tetrapanaxpapyrifer, Vaccariahispanica, Ligusticum striatum, Hedysarumpolybotrys* → *Angelica sinensis* | |
| 48 | *Tetrapanaxpapyrifer, Codonopsispilosula, Glycyrrhiza uralensis → Angelica sinensis* | |
| 49 | *Tetrapanaxpapyrifer, Codonopsispilosula, Glycyrrhiza uralensis, Lyciumchinense → Angelica sinensis* | |
| 50 | *Tetrapanaxpapyrifer, Codonopsispilosula, Glycyrrhiza uralensis, Lyciumchinense, Hedysarumpolybotrys → Angelica sinensis* | |
| 51 | *Tetrapanaxpapyrifer, Codonopsispilosula, Glycyrrhiza uralensis, Hedysarumpolybotrys → Angelica sinensis* | |
| 52 | *Tetrapanaxpapyrifer, Codonopsispilosula, Glycyrrhiza uralensis, Ziziphus jujuba → Angelica sinensis* | |
| 53 | *Tetrapanaxpapyrifer, Codonopsispilosula, Lyciumchinense* → *Angelica sinensis* | |
| 54 | *Tetrapanaxpapyrifer, Codonopsispilosula, Lyciumchinense, Hedysarumpolybotrys → Angelica sinensis* | |
| 55 | *Tetrapanaxpapyrifer, Codonopsispilosula, Ziziphus jujuba, Lyciumchinense → Angelica sinensis* | |
| 56 | *Tetrapanaxpapyrifer, Codonopsispilosula, Ziziphus jujuba, Lyciumchinense, Hedysarumpolybotrys → Angelica sinensis* | |
| 57 | *Angelica sinensis, Cinnamomum cassia → Hedysarumpolybotrys* | |
| 58 | *Rehmanniaglutinosa, Ligusticum striatum, Paeonia lactiflora, Lyciumchinense* → *Hedysarumpolybotrys* | |
| 59 | *Rehmanniaglutinosa, Ligusticum striatum, Paeonia lactiflora, Lyciumchinense* → *Angelica sinensis* | |
| 60 | *Rehmanniaglutinosa, Ligusticum striatum, Paeonia lactiflora, Lyciumchinense, Hedysarumpolybotrys* → *Angelica sinensis* | |
| 61 | *Rehmanniaglutinosa, Ligusticum striatum, Lyciumchinense* → *Angelica sinensis* | |
| 62 | *Rehmanniaglutinosa, Ligusticum striatum, Lyciumchinense, Hedysarumpolybotrys* → *Angelica sinensis* | |
| 63 | *Rehmanniaglutinosa, Ligusticum striatum, Angelica sinensis, Paeonia lactiflora, Lyciumchinense* → *Hedysarumpolybotrys* | |
| 64 | *Rehmanniaglutinosa, Ligusticum striatum, Codonopsispilosula* → *Angelica sinensis* | |
| 65 | *Rehmanniaglutinosa, Vaccariahispanica* → *Angelica sinensis* | |
| 66 | *Rehmanniaglutinosa, Paeonia lactiflora, Lyciumchinense* → *Hedysarumpolybotrys* | |
| 67 | *Rehmanniaglutinosa, Paeonia lactiflora, Lyciumchinense* → *Angelica sinensis* | |
| 68 | *Rehmanniaglutinosa, Paeonia lactiflora, Lyciumchinense, Hedysarumpolybotrys* → *Angelica sinensis* | |
| 69 | *Rehmanniaglutinosa, Lyciumchinense* → *Angelica sinensis* | |
| 70 | *Rehmanniaglutinosa, Lyciumchinense, Hedysarumpolybotrys* → *Angelica sinensis* | |
| 71 | *Rehmanniaglutinosa, Tetrapanaxpapyrifer, Ligusticum striatum, Lyciumchinense* → *Angelica sinensis* | |
| 72 | *Rehmanniaglutinosa, Tetrapanaxpapyrifer, Vaccariahispanica* → *Angelica sinensis* | |
| 73 | *Rehmanniaglutinosa, Tetrapanaxpapyrifer, Lyciumchinense* → *Angelica sinensis* | |
| 74 | *Rehmanniaglutinosa, Tetrapanaxpapyrifer, Lyciumchinense, Hedysarumpolybotrys* → *Angelica sinensis* | |
| 75 | *Rehmanniaglutinosa, Angelica sinensis, Paeonia lactiflora, Lyciumchinense* → *Hedysarumpolybotrys* | |
| 76 | *Rehmanniaglutinosa, Codonopsispilosula* → *Angelica sinensis* | |
| 77 | *Rehmanniaglutinosa, Codonopsispilosula, Lyciumchinense* → *Angelica sinensis* | |
| 78 | *Rehmanniaglutinosa, Codonopsispilosula, Hedysarumpolybotrys* → *Angelica sinensis* | |
| 79 | *Codonopsispilosula, Glycyrrhiza uralensis* → *Angelica sinensis* | |
| 80 | *Codonopsispilosula, Glycyrrhiza uralensis, Lyciumchinense* → *Angelica sinensis* | |
| 81 | *Codonopsispilosula, Glycyrrhiza uralensis, Lyciumchinense, Hedysarumpolybotrys* → *Angelica sinensis* | |
| 82 | *Codonopsispilosula, Glycyrrhiza uralensis, Hedysarumpolybotrys* → *Angelica sinensis* | |
| 83 | *Codonopsispilosula, Glycyrrhiza uralensis, Ziziphus jujuba* → *Angelica sinensis* | |
| 84 | *Codonopsispilosula, Glycyrrhiza uralensis, Ziziphus jujuba, Lyciumchinense* → *Angelica sinensis* | |
| 85 | *Codonopsispilosula, Glycyrrhiza uralensis, Ziziphus jujuba, Lyciumchinense, Hedysarumpolybotrys* → *Angelica sinensis* | |
| 86 | *Codonopsispilosula, Glycyrrhiza uralensis, Ziziphus jujuba, Hedysarumpolybotrys* → *Angelica sinensis* | |
| 87 | *Codonopsispilosula, AtractylodesmacrocephalaKoidz.* → *Angelica sinensis* | |
| 88 | *Codonopsispilosula, Paeonia lactiflora* → *Angelica sinensis* | |
| 89 | *Codonopsispilosula, Lyciumchinense* → *Angelica sinensis* | |
| 90 | *Codonopsispilosula, Lyciumchinense, Hedysarumpolybotrys* → *Angelica sinensis* | |
| 91 | *Codonopsispilosula, Ziziphus jujuba, Lyciumchinense* → *Angelica sinensis* | |
| 92 | *Codonopsispilosula, Ziziphus jujuba, Lyciumchinense, Hedysarumpolybotrys* → *Angelica sinensis* | |
| *Medicinal materials to the right of the arrow would appear in the same galactogenousprescription as the medicinal materials to the left of the arror | | |
